# Community structure, environmental conditions and anthropogenic pressure on the habitat of the European endemic aquatic plant *Luronium natans *(L.) Raf.

**DOI:** 10.1186/s12870-023-04518-y

**Published:** 2023-11-28

**Authors:** Krzysztof Banaś, Rafał Ronowski, Rafał Chmara, Józef Szmeja

**Affiliations:** Department of Plant Ecology, Faculty of Biology, University of Gdansk, Ul. Wita Stwosza 59, 80-308 Gdańsk, PL Poland

**Keywords:** *Luronium* habitats, Aquatic community, Environmental conditions, Anthropogenic pressure

## Abstract

*Luronium natans* (L.) Raf. is a European endemic species and is becoming increasingly rare and endangered in most countries. This study aimed to compare the community structure and environmental conditions of shallow and deep-water habitats of *Luronium*, and related anthropogenic influences. A total of 21 *Luronium* lake habitats were surveyed at Pomerania Lakeland (NW Poland). *Luronium* occurs mainly with other isoetids, as well as bryophytes, specifically *Sphagnum denticulatum*. It can also be found in oligotrophic lakes at a depth of 1.0 ± 0.6 m and in water with a large pH range (4.52 – 8.76), as well having a low conductivity (38.3 ± 20.9 µS cm^−1^; 19.0 – 106.1) and calcium concentration (3.9 ± 2.4 mg dm^−3^; 1.6 – 11,7).

The largest *Luronium* cover occurs at a depth of 1.5 m (44.8 ± 35.3%), but occasionally as deep as 3.5 m. In the depth gradient, the structure of underwater vegetation and environmental conditions exhibit obvious changes, which presents a clear distinction between shallow and deep-water habitats of *Luronium*. The differences mainly pertain to the abundances of *Isoëtes lacustris* and *Elodea canadensis* in the community, as well as environmental factors, such as water calcium, nitrogen and phosphorus concentrations, PAR, conductivity, and water color.

Compared to other isoetids, *Luronium* usually occurs in habitats with intermediate features, which are characterized by values between the typical, but deep-water, *Isoëtes* and shallow water *Lobelia* and *Littorella*. However, *Luronium* clearly prefers waters with higher temperatures (23.8 ± 2.7 °C), which are thus less oxygenated (96.6 ± 20.0%). In terms of pH, conductivity, and calcium concentration, *Luronium* occurs in waters having slightly lower values compared to other isoetids. Therefore, *Luronium* is a species that significantly expands the diversity of habitat number 3110 in the Natura 2000 network. Therefore, it can be considered as an indicator species of lobelia lakes.

An increased anthropogenic pressure primarily results in an increased water conductivity and a decreased water transparency. Consequently, *Luronium* increasingly inhabits shallower waters that are more oxygenated. Moreover, *Luronium* abundance is decreasing, while the abundances of species comprising underwater communities are also decreasing, e.g., *S. denticulatum* and *I. lacustris*, with a concurrent increase in *Myriophyllum alterniflorum* and *E. canadensis* cover.

**Supplementary Information:**

The online version contains supplementary material available at 10.1186/s12870-023-04518-y.

## Background

*Luronium natans* (L.) Raf. (floating water-plantain) is a European endemic species distributed in North-western and Central Europe [1] in the sub-Atlantic and Atlantic climate zones [2–5]. In most European countries, *Luronium* is considered a rare species and in danger of extinction, and in some of them, it is already extinct [6, 7]. It is protected under the Berne Convention, as well as the so-called Habitats Directive within the Natura 2000 Program. Natura 2000 is a network of core breeding and resting sites for rare and threatened species, together with certain rare natural habitat types, which are protected. The Natura 2000 network includes all EU countries, both on land and at sea (European Commission; https://environment.ec.europa.eu/index_en). The Council Directive 92/43/EEC [8], on the conservation of natural habitats, and wild fauna and flora, determined the legal framework for creating the European ecological network Natura 2000.

Floating water-plantain *Luronium natans* (L.) Raf. (synonyms: *Alisma natans* L., *Elisma natans* (L.) Buchenau) is an angiosperm (Angiospermae) and a monocotyledonous species (Monocotyledones) in the Alismataceae plant family. It is a perennial and evergreen plant characterized by clonal growth. Each individual is comprised of unevenly aged ramets connected with stolons. Moreover, it is a rhizophyte and isoetid.

It mainly propagates vegetatively [9], but such vegetative propagation is lower compared to populations of *Lobelia* and *Littorella* [9, 10]. This can result from *Luronium* stolons breaking apart easily in disturbed habitats, and uprooted ramets which fail to root again in the substrate [11]. Offspring ramets fail to obtain assimilates when disconnected from maternal ramets [12]. As with other clonal aquatic plants, the effectiveness of generative propagation is very low [13], and is especially exacerbated in disturbed habitats [14].

*L. natans* is distributed in aquatic and semi-aquatic environments, where it develops into two morphologically distinct forms: underwater and semi-aquatic [15]. The underwater form comprises of a bottom-dwelling plant with a rosette of linear leaves growing on a shortened shoot with a filamentous flowering stem. The leaves in the rosette are ensiform and pointed; however, unlike other isoetids, they are flexible. The root system is of the beam type, while stolons grow from the rosette base. The semi-aquatic form includes shallow-growing individuals, as a nymphaeid plant with long-petioled oval leaves floating on the water surface [16]. This form can be found in several types of aquatic environments, from dystrophic to eutrophic lakes, as well as in anthropogenically transformed reservoirs. To date, the differences in the community structures composed of both forms, as well as their environmental conditions, have not yet been studied.

Literature has demonstrated that the floating water-plantain *L. natans* grows in soft water, but which is highly very variable in terms of pH, ranging from strongly acidic [17–19], slightly acidic, neutral [20–22], and even alkaline [17, 23]. These are most often oligotrophic waters [21, 24–30], and less often mesotrophic [2, 31, 32] or eutrophic [30]. It can be found on mineral substrates which are mainly sandy [2, 17, 18, 30], or a mix of sand and gravel [2, 17], but can be also be found on substrates rich in calcium [27, 33] and organic material [30, 34], such as silt [17, 35] or peat [36, 37]. These substrates are usually moderately fertile [38] and moderately rich in organic matter [2, 18]. Moreover, these are often variable in pH, similar to water, ranging from acidic and neutral, to alkaline [27].

Literature has shown that floating water-plantain is distributed throughout numerous communities of aquatic plants, as well as aquatic and wetland plants [22, 32, 39–42]. It is firstly a component of the *Littorelletea uniflorae* Br.-Bl. et R.Tx. 1943 class communities [21, 42, 43]. However, it is not yet clear with which isoetids it coexists most frequently, and whether it occurs in the same or different habitats from other isoetids.

In most countries, *L. natans* is an increasingly rare and endangered species. England is an exception, since this species, although disappearing, has not yet been classified as endangered [6, 44]. The extinction of *L. natans* populations is significant, but more thorough underwater research, as well as the discovery of new habitats where they occur (e.g., dykes, ditches, equalization ponds, water reservoirs formed due to mineral extraction and peat mines) mean that the total number of known populations have not yet decreased drastically [6]. Previous studies have shown that *L. natans* is a species with a high phenotypic plasticity, specifically carbon acquisition plasticity, and can adapt to different habitat conditions [33, 45].

The following hypotheses are presented here:

1. The community structure of *Luronium* differs according to water gradient.
2. The environmental conditions of *Luronium* differ according to the water gradient.
3. *Luronium natans* has a broader ecological spectrum than other isoetids found in softwater lobelia lakes (habitat 3110); Oligotrophic waters containing very few minerals of sandy plains (*Littorelletalia uniflorae*).
4. A slight increase in anthropogenic pressure leads to an increase in *Luronium* abundance. In turn, the strong anthropogenic pressure on *Luronium* habitats affects environmental conditions and community structure. Additionally, *Luronium* is found in increasingly shallower habitats, in decreasing abundance, and in communities with decreasing species numbers.

The aim of this study was to compare the community structures and environmental conditions of shallow and deep-water habitats of *L. natans* subjected to anthropogenic pressures of varying intensities. Moreover, the study aimed to identify the main differences in *Luronium* habitats as compared to habitats of other isoetids that are found in the lakes of north-western Poland.

## Results

### Structure of *Luronium natans* community

**Fig. 1:**
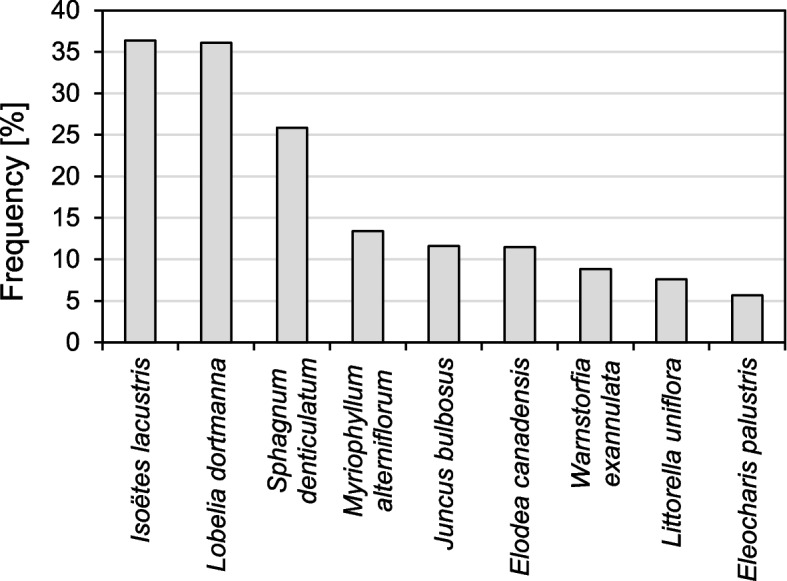
Frequency of species in the *Luronium natans* community

A total of 38 plant species were found in the *L. natans* community, including a large group of helophytes (11 species). On average, there were 3.3 ± 1.7 species per 0.1 m^2^ (1–10, Me = 4). Only 9 species had a frequency exceeding 5%, while 6 species had a frequency exceeding 10%. *Isoëtes lacustris* had the highest frequency in the community (36.4%), with *Lobelia dortmanna* being similar (36.1%), and *Sphagnum denticulatum* also having a relatively high frequency (25.9%). The other species were much less frequent (Table 1; Fig. 1).

**Table 1:** Frequency (F) and species abundance in the *Luronium natans* community

Species	No. of samples	F [%]	Coverage [%]				
			Mean	SD	Min	Max	Median
*Chara delicatula* Agardh	3	0.4	1.0	0.0	1	1	1
*Chara globularis* J.L.Thuiller	31	4.3	3.6	2.4	1	9	3
*Nitellopsis obtusa* (Desvaux in Loiseleur-Deslongchamps) J. Groves	1	0.1	1.0		1	1	1
*Drepanocladus sordidus* T. Kop.	2	0.3	1.5	0.7	1	2	1.5
*Fontinalis antipyretica* Hedw.	3	0.4	2.0	1.0	1	3	2
*Fontinalis dalecarlica* Schimp.	2	0.3	1.0	0.0	1	1	1
*Sphagnum denticulatum* Brid.	187	25.9	18.4	15.7	1	80	15
*Sphagnum fallax* (H.Klinggr.) H.Klinggr.	6	0.8	3.3	1.6	1	5	3.5
*Warnstorfia exannulata* (Schimp.) Loeske	64	8.9	16.8	27.1	1	100	5
*Carex lasiocarpa* Ehrh.	16	2.2	1.8	0.8	1	3	2
*Carex rostrata* Michx.	11	1.5	2.5	1.4	1	5	
*Carex elata* All.	4	0.6	2.5	1.3	1	4	2.5
*Ceratophyllum demersum* L.	1	0.1	2.0		2	2	2
*Eleocharis palustris* (L.) Roem. & Schult.	41	5.7	9.6	8.1	1	30	5
*Elodea canadensis* Michx.	83	11.5	15.2	12.0	1	55	15
*Equisetum fluviatile* L.	4	0.4	1.0	0.0	1	1	1
*Hydrocotyle vulgaris* L.	4	0.6	2.0	0.8	1	3	2
*Isoëtes lacustris* L.	263	36.4	35.7	29.3	1	100	25
*Juncus bulbosus* L.	84	11.6	6.7	2.9	1	12	7
*Juncus effusus* L.	1	0.1	1.0		1	1	1
*Littorella uniflora* (L.) Asch.	55	7.6	13.6	14.9	1	60	5
*Lobelia dortmanna* L.	261	36.1	25.8	20.8	1	100	20
*Luronium natans* (L.) Raf.	723	100.0	35.3	30.6	1	100	25
*Lysimachia thyrsiflora* L.	8	1.1	4.9	1.4	2	7	5
*Myriophyllum alterniflorum* DC.	97	13.4	17.9	17.9	1	90	10
*Nuphar lutea* (L.) Sibth. & Sm.	3	0.4	1.0	0.0	1	1	1
*Nymphaea alba* L.	3	0.4	2.0	1.0	1	3	2
*Polygonum amphibium* L.	2	0.3	1.5	0.7	1	2	1,5
*Potamogeton x angustifolius* J. Presl	2	0.3	1.0	0.0	1	1	1
*Potamogeton compressus* L.	1	0.1	1.0		1	1	1
*Potamogeton natans* L.	4	0.6	1.8	1.0	1	3	1.5
*Potamogeton obtusifolius* Mert. & W.D.J. Koch	2	0.3	1.5	0.7	1	2	1.5
*Ranunculus reptans* L.	3	0.4	1.3	0.6	1	2	1
*Sparganium angustifolium* Michx.	5	0.7	1.6	0.5	1	2	2
*Typha latifolia* L.	1	0.1	10.0		10	10	10
*Lycopus europaeus* L.	1	0.1	1.0		1	1	1
*Batrachospermum* Roth	34	4.7	10.6	7.2	3	30	10
*Betula pendula* Roth	1	0.1	1.0		1	1	1
Number of species/0.1m^2^			3.3	1.7	1	10	4

**Fig. 2:**
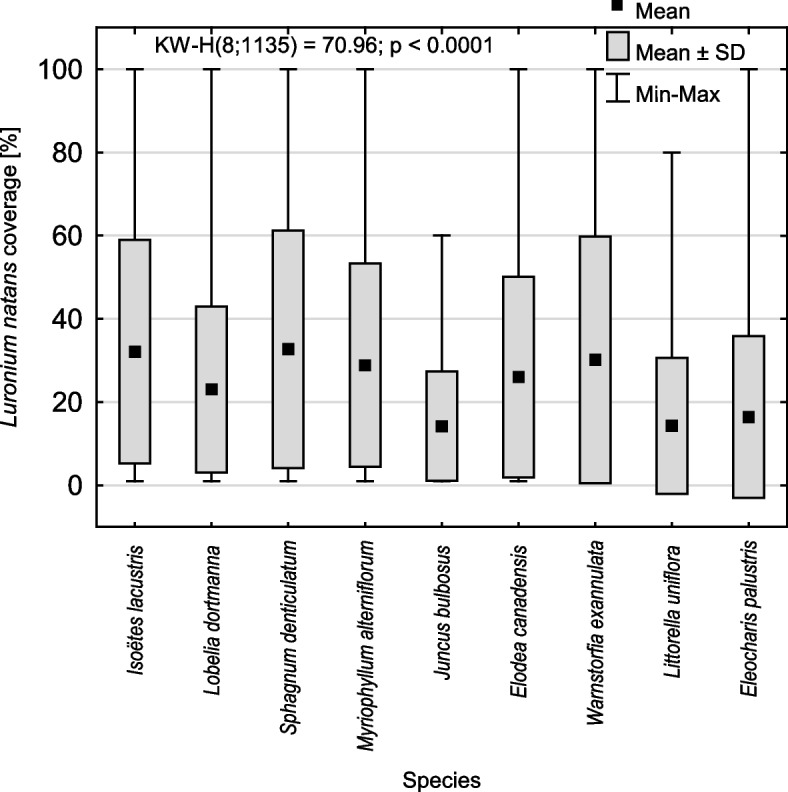
*Luronium natans* cover in patches with a species frequency of > 5%

*Isoëtes lacustris* as observed in the community also had the highest abundance (cover of 35.7 ± 29.3%; 1–100%; Me = 25%), with *L. natans* having only slightly less cover (35.3 ± 30.6%; 1–100%; Me = 25%). A high abundance was also found for *L. dortmanna* (25.8 ± 20.8%; 1–100%; Me = 20%), while significantly lower abundances were found for *S. denticulatum*, *Myriophyllum alterniflorum*, *Warnstorfia exannulata*, *Elodea canadensis* and *L. uniflora* (Table 1). The highest *Luronium* abundance was observed in patches composed with *I. lacustris* and *S. denticulatum*, whereas the lowest abundance occurred in patches with *L. uniflora* and *Juncus bulbosus* (Fig. 2).

The vertical structure of the *Luronium* community was poorly developed. Specifically, it was a monolayered community dominated by isoetids and *Sphagnum*. However, elodeids were also found in it, mainly *M. alterniflorum* and *E. canadensis*. These two species comprised the vertical structure, and their percentage frequency exceeded about 12%. Similar results were observed for average cover, but on occasion they were found in community patches with up to 90% cover (Table 1). In contrast, the proportion of rush plants (*Eleocharis palustris*, *Juncus effusus*, *J*. *bulbosus* and *Lysimachia thyrsiflora*) was small, and their frequencies and covers had small percentage values. The vertical structure was also occasionally formed by nymphaeids (Table 1). The spatial variability of the community was small and mainly related to depth gradient changes, and only at community edges did *Luronium* have a small cover (< 5%), in contrast to the other isoetids or *S. denticulatum*. This variability was noticeable primarily in the depth gradient (cf. section on the “Shallow- and deep-water populations of *L. natans*”).

### Environmental conditions

*Luronium natans* habitats occurred at a depth of 1.0 ± 0.6 m (Me = 0.5) in water with a very different pH (4.52 – 8.76; Me = 5.88), and characterized by a low conductivity (38.3 ± 20.9 µS cm^−1^; 19.0 – 106.1) and calcium concentration (3.9 ± 2.4 mg dm^−3^; 1.6 – 11,7). Moreover, the water was clear and well oxygenated, but significantly colored by humic acids (Table 2). The sediment within the community was characterized by acidic reactions and, similar to the water, also had a low conductivity and calcium concentration. It was also rich in mineral content and poorly hydrated.

**Table 2:** Environmental conditions of *Luronium natans*

	N	Mean	SD	Min	Max	Median
water characteristics						
pH	723	-	-	4.52	8.76	5.88
Eh [mV]	192	142.3	89.1	5	419	94
Conductivity [µS cm^−1^]	723	38.3	20.9	19.0	106.1	28.1
Ca [mg dm^−3^]	723	3.9	2.4	1.6	11.7	2.9
CO_2_ [mg dm^-3^]	147	3.39	2.73	0	9.42	2.3
HCO_3_^-^ [mg dm^-3^]	246	13.96	10.81	6.86	91.08	11.44
P_tot._ [mg dm^−3^]	714	0.05	0.04	0.01	0.22	0.04
N_tot._ [mg dm^−3^]	714	0.90	0.38	0.27	2.50	0.76
Water color [mg Pt dm^−3^]	723	31.8	24.9	5	130	22
Humic acids [mg dm^−3^]	723	2.6	1.6	0.8	9.3	2.5
Oxygenation [%]	616	96.6	20.0	37.2	155.7	91.5
Temperature [^o^C]	616	23.8	2.7	18.8	27.8	24.4
PAR [%]	713	25.8	15.7	0.7	86.7	23.1
Water transparency[m]	723	3.3	1.1	1.0	7.0	3.4
Depth [m]	723	1.0	0.6	0.5	3.5	0.5
sediment characteristics						
pH	307	-	-	4.20	6.85	5.90
Eh [mV]	307	-80.3	157.0	-276	314	-77
Conductivity [µS cm^−1^]	307	38.5	24.9	6.7	144.7	29.3
Organic matter [%]	307	5.1	14.0	0.3	63.4	1.0
Hydration [%]	307	29.8	15.8	4.5	94.1	25.2
Mineral matter [%]	307	92.7	17.0	36.6	99.7	99.3

*Luronium* communities primarily occurred in waters having the highest (slightly alkaline) pH, together with *M. alterniflorum* and *E. canadensis*, whereas acidic waters were characterized by *S. denticulatum*, *W. exannulata*, *I. lacustris*, and *Eleocharis palustris* (see Additional file 1: Fig. S1). *Luronium*, as well as *W. exannulata*, *S. denticulatum*, *J. bulbosus* and *E. palustris*, also occurred in habitats with the highest water redox potential, while in low redox habitats were characterized by *M. alterniflorum*, *E. canadensis*, *I. lacustris,* and *L. dortmanna*. *Myriophyllum alterniflorum* and *E. canadensis* co-occurred with *Luronium* in waters rich in calcium, whereas other species were found in waters with low Ca^2+^ levels; these mainly included *S. denticulatum* and *W. exannulata*. *Sphagnum denticulatum* and *W. exannulata* also occurred concurrently with *Luronium* in significantly colored habitats, whereas *I. lacustris*, *M. alterniflorum* and *E. canadensis* were mainly found in less colored waters.

Most species coexisted with *Luronium* in very shallow waters (at a depth of ca. 0.5 m), while *W. exannulata* and *I. lacustris* mainly occurred in deeper zones (ca. 1.5–3.5 m; see Additional file 1:  Fig. S1).

*Myriophyllum alterniflorum* and *E. canadensis*, together with *Luronium*, grew mainly in waters with the highest oxygen levels, while other species were found in less oxygenated waters. *Warnstorfia exannulata* and *S. denticulatum* were specifically found in the least oxygenated waters (see Additional file 1: Fig. S2). *Eleocharis palustris* was found together with *Luronium* in the warmest and most illuminated waters, whereas colder waters were mainly characterized by *J. bulbosus*, and deep-water species such as *W. exannulata*, occurred in poorly lit waters.* Isoёtes lacustris* occurred mainly in waters with the highest transparency, together with *Luronium*, and waters with the lowest transparency were characterized by *E. palustris* and *J. bulbosus*.

*Littorella uniflora*, *E. canadensis*, and *M. alterniflorum*, together with *Luronium*, grew in sediments with the highest pH (see Additional file 1: Fig. S3), whereas sediments with the lowest pH (strongly acidic) were characterized mainly by *W. exannulata* and *S. denticulatum*, as well as *E. palustris*. Sediments with the highest electrolytic conductivity, organic matter density, and hydration levels were characterized by *Luronium* assemblages with *W. exannulata*. Sediment features failed to significantly differentiate the habitats of other *L. natans* assemblages.

**Fig. 3:**
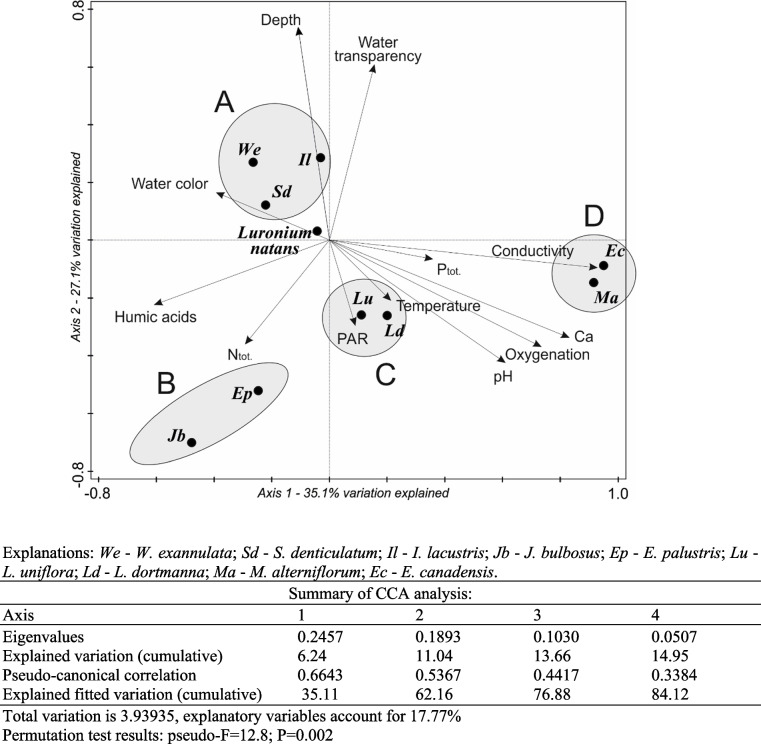
Environmental factor ranking in the formation of *Luronium* community groups (A-D; CCA method)

In conclusion: *Luronium* habitats were very similar in terms of environmental conditions compared to *S. denticulatum* and *I. lacustris*, as well as *W. exannulata* (group A; Fig. 3). *Luronium* assemblages that had these species in common were mainly found in medium depth phytolitoral zones, and in acidic waters that were poor in calcium, light, and oxygen levels, but that were significantly colored. *Lobelia dortmanna* and *L. uniflora* (group C) were distributed with *Luronium* mainly in shallow waters with a higher pH than group A, as well as being well lit and oxygenated, while being poorly colored. *Eleocharis palustris* and* J. bulbosus* (group B) were also found in shallow water habitats that were rich in nitrogen and humic acids, and where water transparency was very low. significantly with the depth gradient*. canadensis* (group D; Fig. 3) occurred together with *Luronium* mainly in waters with a high electrolytic conductivity, and that were rich in calcium and phosphorus, well oxygenated, very poorly colored and with high pH levels.

### Shallow- and deep-water populations of *L. natans*

**Fig. 4:**
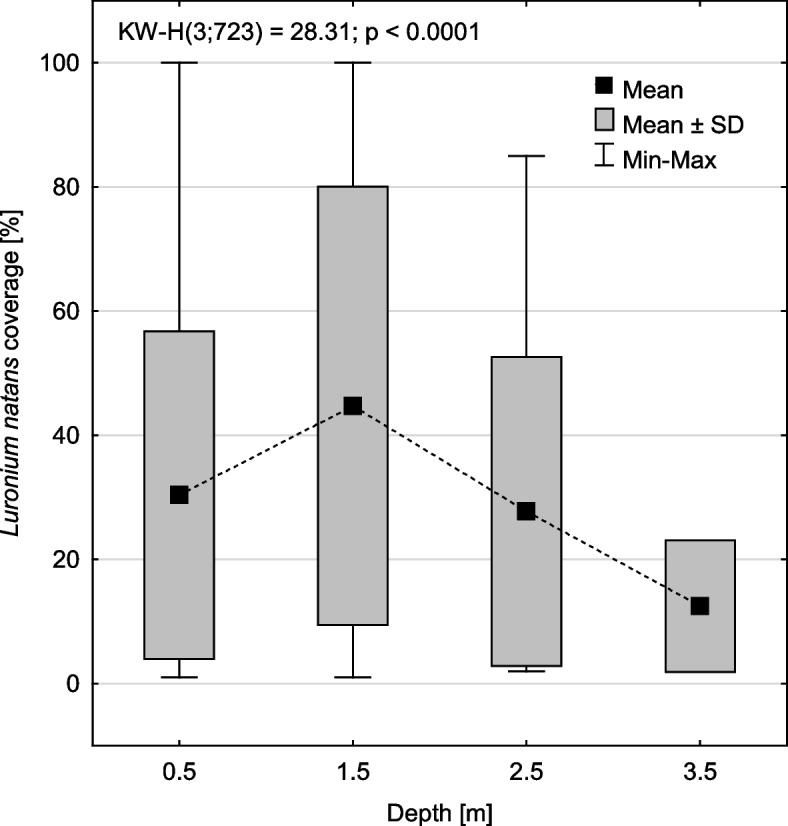
Changes in *Luronium* cover in relation to water depth

*Luronium natans* was found in lakes at a depth of up to 3.5 m, and its abundance changed significantly with the depth gradient (*p* < 0.0001; Fig. 4). The largest cover occurred at a depth of 1.5 m (44.8 ± 35.3%), and was larger as compared to the shallow zone 0.5 m (*p* < 0.0001; RIR Tukey test for unequal numbers) and deep zone at 2.5 m (*p* = 0.037; Fig. 4, Table 3). *Luronium* was only occasionally found in the deepest zone (3.5 m). Vegetation structure and environmental conditions changed evidently in relation to the depth gradient for *Luronium* habitats. The shallow-water population was found up to a depth of 1 m (average depth of 0.5 m), which was different compared to the deep-water population that was characterized by a range of 1–4 m (average depth of 2.5 m) and the occurrence of 24 plant species. Only 14 species were common to both populations (Table 4).

**Table 3:** Differences in abundance of *L. natans* in the depth zones

Depth [m]	N	Coverage [%]					*p* for HSD Tukey test for unequal N			
		Mean	SD	Min	Max	Median	Depth [m]	1.5	2.5	3.5
0.5	420	30.4	26.4	1	100	20	0.5	** < 0.0001**	0.98	0.93
1.5	257	44.8	35.3	1	100	30	1.5		**0.037**	0.70
2.5	44	27.8	24.9	2	85	20	2.5			0.96
3.5	2	12.5	10.6	5	20	12.5	3.5			
Total	723	35.3	30.6	1	100	25

**Table 4:** Species coverage in patches of shallow- (zone 1) and deep-water (zone 2) habitats of *L. natans*. Statistically significant differences between zones are shown in bold (Mann–Whitney U test with *p* < 0.05)

	Zone 1 0–1 m (0.5 m)			Zone 2 1–4 m (2.5 m)			Mann–Whitney U test		
Species	N	Mean	SD	N	Mean	SD	U	Z	p
*Chara delicatula*	3	1.0	0.0	0					
*Chara globularis*	14	2.5	1.2	17	4.6	2.2			
*Nitellopsis obtusa*	1	1.0		0					
*Drepanocladus sordidus*	0			2	1.5	0.7			
*Fontinalis antipyretica*	0			3	2.0	1.0			
*Fontinalis dalecarlica*	2	1.0	0.0	0					
*Sphagnum denticulatum*	112	19.9	18.5	75	16.2	9.7	4155.5	0.12	0.902
*Sphagnum fallax*	1	1.0		5	2.4	1.1			
*Warnstorfia exannulata*	21	19.1	28.0	43	15.6	26.9	328.0	1.79	0.074
*Carex lasiocarpa*	16	1.8	0.8	0					
*Carex rostrata*	11	2.5	1.4	0					
*Carex elata*	4	2.5	1.3	0					
*Ceratophyllum demersum*	1	2.0		0					
*Eleocharis palustris*	35	10.3	8.6	6	5.0	0.0	72.0	1.28	0.201
***Elodea canadensis***	**65**	**13.4**	**11.2**	**18**	**21.9**	**13.0**	**332.0**	**-2.83**	**0.005**
*Equisetum fluviatile*	4	1.0	0.0	0					
*Hydrocotyle vulgaris*	4	2.0	0.8	0					
***Isoëtes lacustris***	**99**	**21.4**	**18.9**	**164**	**44.3**	**31.1**	**4417.00**	**-6.22**	** < 0.001**
*Juncus bulbosus*	49	7.4	3.2	35	2.6	0.8			
*Juncus effusus*	1	1.0		0					
*Littorella uniflora*	47	14.8	15.8	8	6.3	2.3	142.00	1.13	0.257
***Lobelia dortmanna***	**227**	**27.2**	**21.7**	**34**	**16.5**	**9.3**	**2824.50**	**2.53**	**0.011**
***Luronium natans***	**420**	**30.4**	**26.4**	**303**	**42.1**	**34.4**	**52,371.00**	**-4.08**	** < 0.001**
*Lysimachia thyrsiflora*	8	4.9	1.4	0					
*Myriophyllum alterniflorum*	71	16.6	17.8	26	21.4	18.3	724.00	-1.64	0.101
*Nuphar lutea*	3	1.0	0.0	0					
*Nymphaea alba*	0			3	2.0	1.0			
*Polygonum amphibium*	2	1.5	0.7	0					
*Potamogeton x angustifolius*	0			2	1.0	0.0			
*Potamogeton compressus*	0			1	1.0				
*Potamogeton natans*	4	1.8	1.0	0					
*Potamogeton obtusifolius*	1	1.0		1	1.0				
*Ranunculus reptans*	3	1.3	0.6	0					
*Sparganium angustifolium*	5	1.6	0.5	0					
*Typha latifolia*	1	10.0		0					
*Lycopus europaeus*	1	1.0		0					
***Batrachospermum***	**2**	**3.0**	**0.0**	**32**	**11.1**	**7.2**	**2.00**	**-2.21**	**0.027**
*Betula pendula*	1	1.0		0					
Number of species	420	2.9	1.1	303	3.1	1.7			

**Fig. 5:**
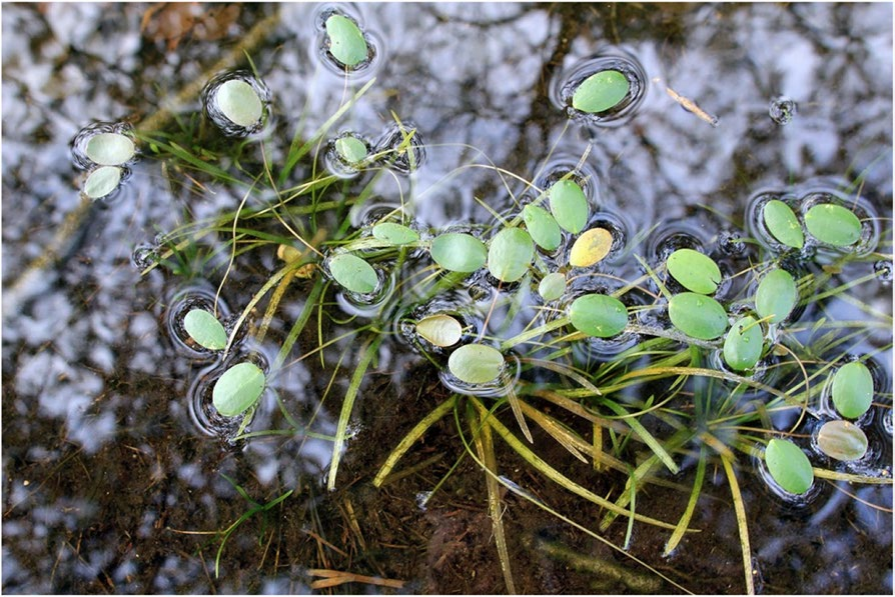
Shallow-water *L. natans* individuals with a characteristic and substantial number of floating leaves

In shallow-water habitats (0–1 m), *L. natans* usually produced numerous floating leaves (Fig. 5), whereas underwater rosettes were poorly developed. *Luronium* occurred with 30.4 ± 26.4% (1–100%) cover, and the community had 34 additional species. However, most of these were not very frequent (Table 4). Only 9 species had a frequency above 5%, while 7 had a frequency above 10% (*L. dortmanna* 54.0%, *S. denticulatum* 26.7%, *I. lacustris* 23.6%, *M. alterniflorum* 16.9%, *E. canadensis* 15.5%, *J. bulbosus* 11.7%, *L. uniflora* 11.2%). The largest *Luronium* cover occurred with *L. dortmanna* (27.2 ± 21.7%) and *I. lacustris* (21.4 ± 18.9%) in shallow-water habitats, while *S. denticulatum* (19.9 ± 18.5%) and *W. exannulata* (19.1 ± 28.0%) also had significant abundances. The *Luronium* shallow-water habitats were characterized by a lightly acidic water pH (4.52–8.76; Me = 6.01), a low conductivity and calcium concentration, as well as significant PAR intensity and a high oxygen level. The sediments in this zone were mineral, and were poorly hydrated and strongly reduced (Table 5).

**Table 5:** Environmental conditions of the shallow- (zone 1) and deep-water (zone 2) habitats of *L. natans*. Statistically significant differences between zones are shown in bold (Mann–Whitney U test with *p* < 0.05)

	Zone 1 0–1 m (0.5 m)			Zone 2 1–4 m (2.5 m)			Mann–Whitney U test		
	N	Mean	SD	N	Mean	SD	U	Z	p
water characteristics									
** Eh [mV]**	**120**	**110.4**	**82.1**	**72**	**195.4**	**74.1**	**2130.5**	**-5.89**	** < 0.001**
** Conductivity [µS cm**^**−1**^**]**	**420**	**42.8**	**24.0**	**303**	**32.0**	**13.4**	**50,705.5**	**4.67**	** < 0.001**
** Ca [mg dm**^**−3**^**]**	**420**	**4.6**	**2.7**	**303**	**3.0**	**1.6**	**37,055.0**	**9.60**	** < 0.001**
** CO**_**2**_** [mg dm**^**-3**^**]**	**59**	**3.55**	**3.02**	**88**	**2.70**	**0.30**	**45.00**	**-0.59**	**0.553**
**HCO**_**3**_^**-**^ **[mg dm**^**-3**^**]**	**97**	**17.40**	**16.21**	**149**	**11.62**	**2.38**	**4290.50**	**1.73**	**0.084**
** P**_**tot.**_** [mg dm**^**−3**^**]**	**411**	**0.06**	**0.03**	**303**	**0.05**	**0.04**	**42,747.5**	**7.18**	** < 0.001**
** N**_**tot.**_** [mg dm**^**−3**^**]**	**411**	**1.02**	**0.42**	**303**	**0.73**	**0.24**	**32,011.0**	**11.14**	** < 0.001**
** Color [mg Pt dm**^**−3**^**]**	**420**	**27.5**	**17.2**	**303**	**37.7**	**31.7**	**57,838.0**	**-2.11**	**0.035**
Humic acids [mg dm^−3^]	420	2.6	1.3	303	2.6	1.9	58,313.5	1.92	0.055
** Oxygenation [%]**	**330**	**103.7**	**19.2**	**286**	**88.3**	**17.7**	**26,413.0**	**9.46**	** < 0.001**
** Temperature [ºC]**	**330**	**24.1**	**2.8**	**286**	**23.3**	**2.4**	**39,738.0**	**3.40**	**0.001**
** PAR [%]**	**410**	**34.1**	**14.8**	**303**	**14.6**	**8.0**	**14,127.0**	**17.70**	** < 0.001**
Water transparency [m]	420	3.25	1.03	303	3.48	1.29	63,176.5	-0.17	0.869
** Depth [m]**	**420**	**0.5**	**0.0**	**303**	**1.7**	**0.4**	**0.0**	**-26.36**	** < 0.001**
sediment characteristics									
** Eh [mV]**	**218**	**-100.0**	**151.9**	**89**	**-32.1**	**160.0**	**6791.0**	**-4.13**	** < 0.001**
Conductivity [µS cm^−1^]	218	35.9	22.0	89	44.7	30.2	8552.0	-1.63	0.103
** Organic matter [%]**	**218**	**2.3**	**6.9**	**89**	**12.05**	**22.25**	**5159.0**	**-6.44**	** < 0.001**
** Hydration [%]**	**218**	**24.9**	**5.8**	**89**	**41.57**	**24.16**	**4519.0**	**-7.35**	** < 0.001**
** Mineral matter [%]**	**128**	**96.8**	**8.9**	**72**	**85.33**	**24.02**	**1220.0**	**8.64**	** < 0.001**

**Fig. 6:**
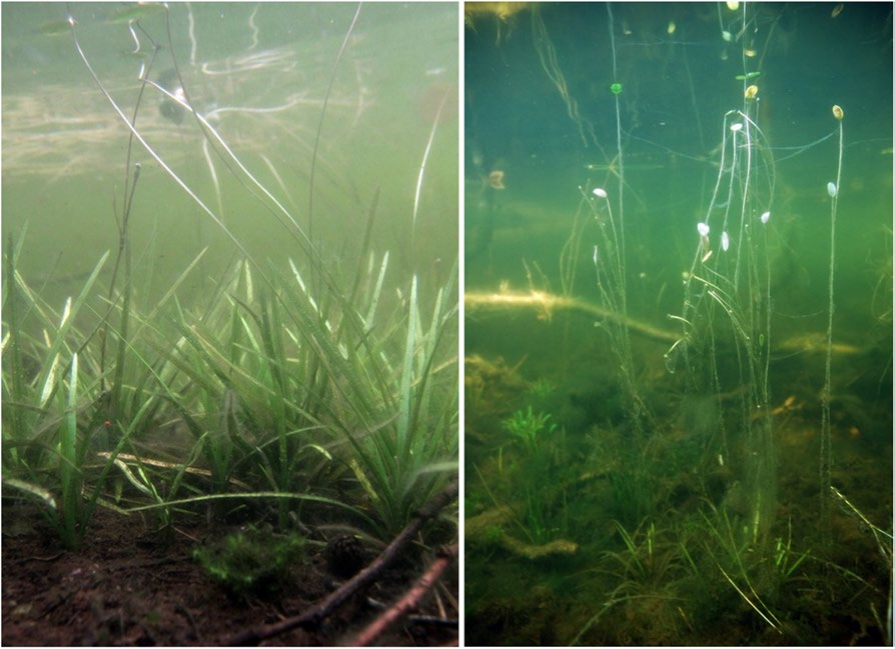
Underwater rosettes composed of ensiform leaves, which are characteristic of deep-water *L. natans* habitats

In deep-water habitats (1–4 m), *Luronium* specimens were characterized by very well-developed underwater rosettes and a low number of floating leaves, which had very long and delicate petioles (Fig. 6). Such habitats occurred at a depth of 1.7 ± 0.4 m and were characterized by a much higher *L. natans* cover of 42.1 ± 34.4% (*p* < 0.001) compared to shallow-water zones. There were much fewer accompanying species (20; Table 4), with 9 species featuring a frequency of over 5% and 6 above 10% per 0.1 m^2^. *Isoëtes lacustris* had nearly double the cover of other species (44.3 ± 31.1%; *p* < 0.001), with *E. canadensis* also having a high cover of 21.9 ± 13.0 (*p* = 0.005), together with *Batrachospermum* (11.1 ± 7.2%; *p* < 0.027), while *L. dortmanna* (16.5 ± 9.3%; *p* = 0.011) had a low cover. Deep-water habitats were poorer in species compared to shallow-water zones. Specifically, these were marked by a lower pH (4.75–7.35; Me = 5.62; *p* < 0.001), conductivity, lower calcium, phosphorus and nitrogen (*p* < 0.001) concentrations, and low oxygen levels and PAR intensities (*p* < 0.001), whereas water color (*p* = 0.035) and its redox potential (*p* < 0.001) were higher, as well as sediment hydration, organic matter content, and sediment redox potential (*p* < 0.001; Table 5).

**Fig. 7:**
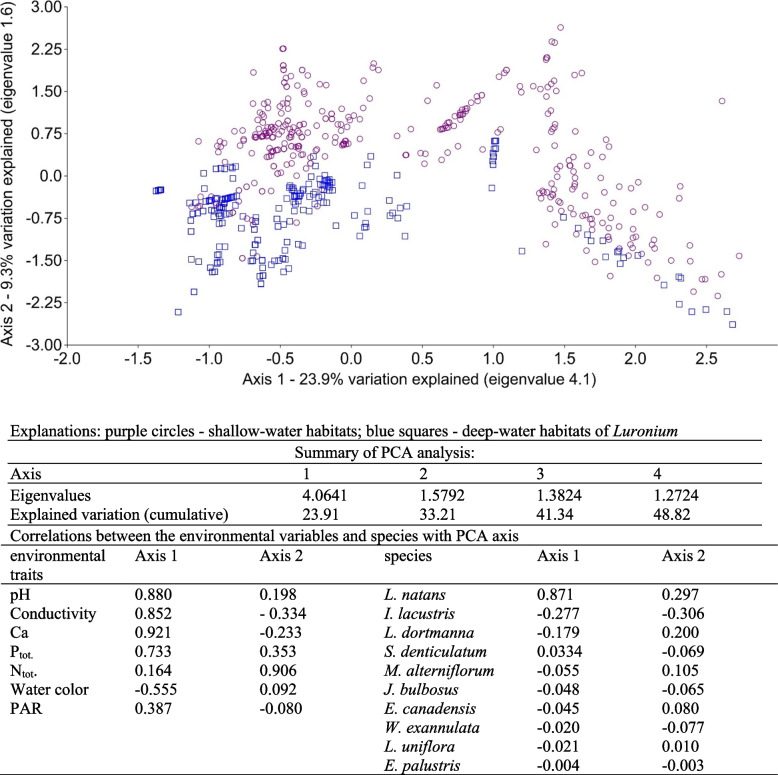
Distribution of *Luronium natans* habitats resulting from PCA analysis, including species cover and environmental conditions

Considering the community structures and the features of the aquatic environments, the shallow-water habitat of *Luronium* occurred in a different part of the PCA graph compared to deep-water zones (Fig. 7). Although sample diversity was larger along the first axis (due to environmental conditions), the separation of shallow and deep-water habitats was mainly determined by features correlated with the second axis. These primarily included positively correlated water nitrogen (0.91) and phosphorus (0.35) concentrations, and negatively correlated abundances of *I. lacustris* (-0.31). Habitats diversities along the first axis were determined by environmental conditions. The strongest positive correlations with this axis were calcium concentration (0.92), pH (0.88), conductivity (0.85), phosphorus concentration (0.73), while water color (-0.56) was negatively correlated.

### *Luronium natans* versus other isoetids

The average depth of *Luronium* was 1.0 ± 0.6 m (Me = 0.5 m) and was greater compared to shallow-water species, such as *L. dortmanna* and *L. uniflora*, and much smaller than the deep-water *I. lacustris* (see Additional file 1: Fig. S4). Moreover, in terms of PAR radiation intensity, *Luronium* revealed indirect features compared to other isoetids, as it occurred in waters with a higher PAR than *I. lacustris*, and a lower than *L. dortmanna* and *L. uniflora*. However, compared to other isoetids, *Luronium* preferred waters with a higher temperature (23.8 ± 2.7 °C) and that were less oxygenated (96.6 ± 20.0%). The values of these traits were associated with higher concentrations of dissolved organic matter, as indicated by the strongest water color (32 ± 25 mg Pt dm^−3^) and were thus characterized by the poorest water transparency (3.3 ± 1.1 m). Moreover, *L. natans* occurred in waters with the lowest pH, conductivity, and calcium concentration values, while in terms of redox potential, it occurred in habitats similar to *I. lacustris*, with a slightly higher potential than *L. dortmanna*, and a lower potential than *L. uniflora* (see Additional file 1: Fig. S5). *Luronium natans* also occurred in habitats that were characterized by a very low concentration of nitrogen and phosphorus (see Additional file 1: Fig. S6). In *Luronium* habitats, mean carbon dioxide concentration was 3.39 ± 2.73 mg CO_2_ dm^−3^ (0.0–9.42; Me = 2.30), and does not differ from the values found in the habitats of the other isoetids (Additional file 1: Fig. S8). Mean bicarbonate concentration in *Luronium* habitats was 13.96 ± 10.81 mg HCO_3_^−^ dm^−3^ (6.86–91.08; Me = 11.44), and were the same as in *I. lacustris* and *L. dortmanna* habitats. However, the bicarbonate concentration found in *Littorella* habitats stood out, as it was higher than the habitats of all isoetids with *p* < 0.0001. Isoetid habitats differed slightly in terms of sediment features. *Luronium* was found in sediments similar to those preferred by *L. dortmanna* and *L. uniflora*, but that were poorer in organic matter and distinguished by a lower redox potential (see Additional file 1: Figs. S7, S8).

**Fig. 8:**
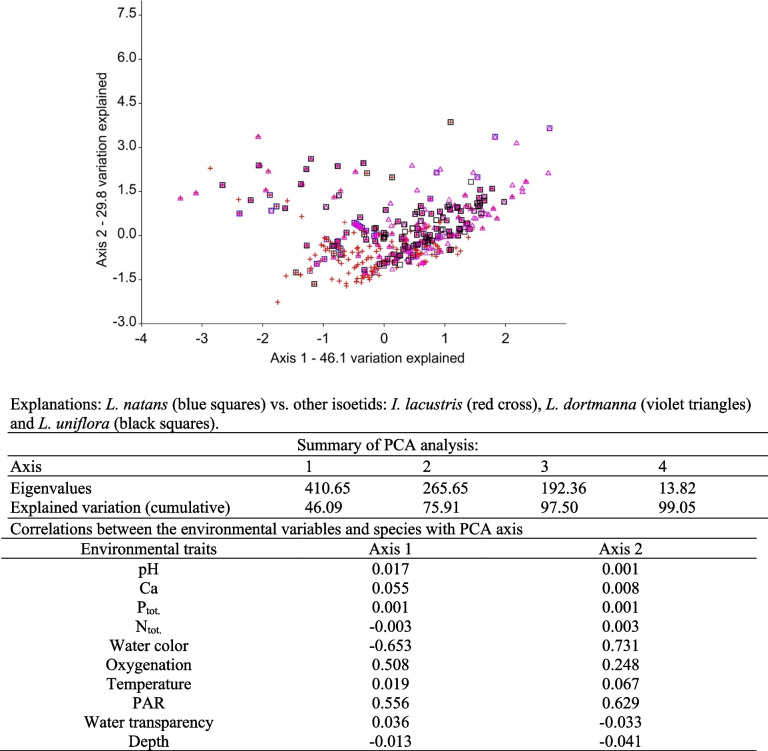
PCA analysis based on species presence-absence values and environmental variables

The PCA analysis showed that the *L. natans* community did not differ from other isoetids (Fig. 8) when accounting for the occurrence of other species and environmental conditions. It should therefore be assumed that they occurred in one habitat and formed one community. The diversity of isoetid habitats along the first axis was primarily determined by water color (-0.65), PAR intensity (0.56), and water oxygenation (0.51). The diversity of habitats along the second PCA axis was determined by positively correlated environmental characteristics, such as water color (0.73), PAR intensity (0.63), and water oxygenation (-0.25).

### Anthropogenic pressure on *Luronium* habitats

The increase in the intensity of anthropogenic pressure significantly limited the depth range of the aquatic plants analyzed, but also increased the number of existing species (Table 6). However, no significant correlation was found between pressure intensity and *Luronium* cover, as well as general underwater plant cover. Importantly, pressure impacts that were directed at the lake and its catchment were very similar.

**Table 6:** Spearman’s rank correlation (marked correlation coefficients represent *p* < 0.05)

	Depth	No. of species	*L. natans* coverage	Total plant coverage
pressure at the lake	**-0.331**	**0.274**	-0.013	0.066
pressure at the catchment	**-0.358**	**0.246**	-0.011	0.013
total pressure	**-0.337**	**0.263**	-0.011	0.051

**Fig. 9:**
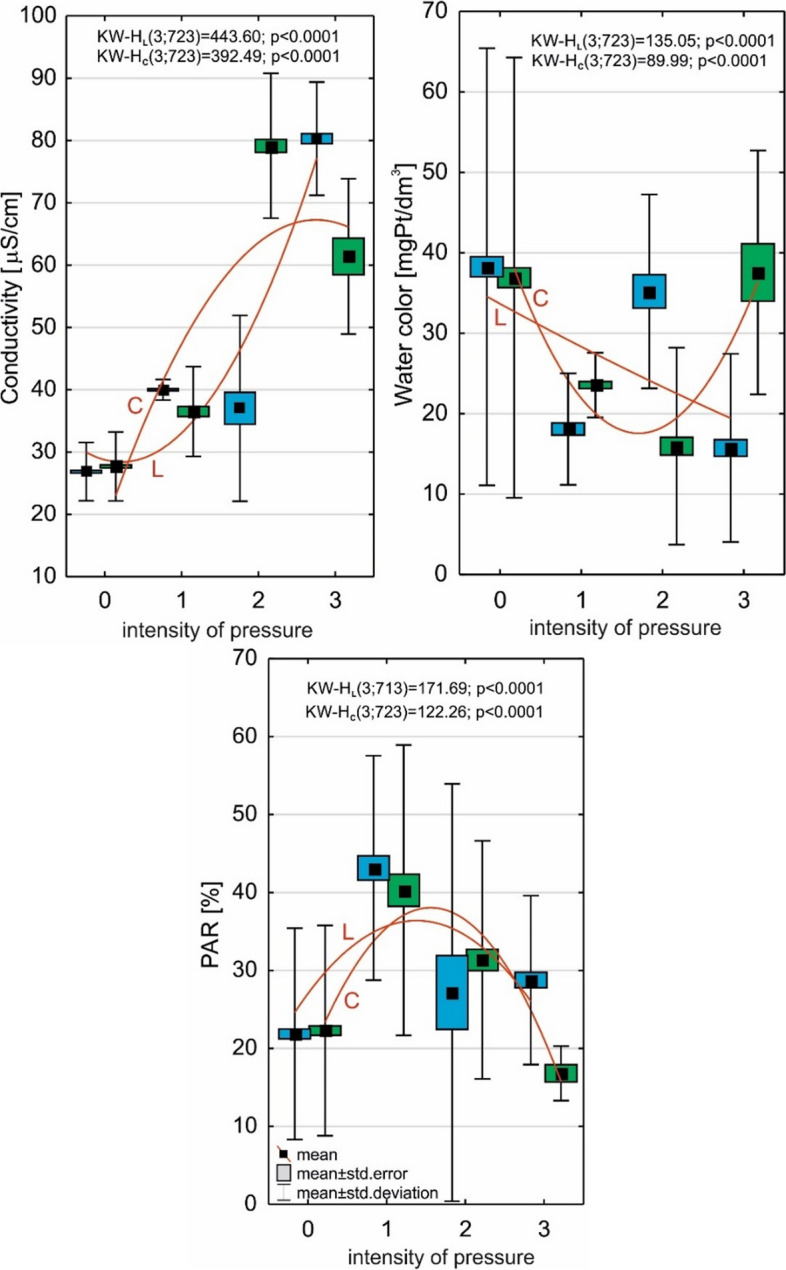
The impact of the intensity of anthropogenic pressure on *L. natans* habitats. Explanations: L; blue color—the impact of the intensity of anthropogenic pressure on the lake; C; green color—the impact of the intensity of anthropogenic pressure on the catchment area;—conductivity (respectively L and C: y = 44.37–21.94x + 7.53x^2^; y = -18.27 + 47.71x-6.66x^2^);—water color (respectively L and C: y = 40.34–5.95x + 0.18x^2^; y = 73.58–44.24x + 8.74x^2^);—PAR intensity (respectively L and C: y = 4.45 + 25.08x-4.92x^2^; y = -6.73 + 38.19x-8.15x.^2^)

Anthropogenic pressure directed at *Luronium* habitats resulted in a clear transformation of its environmental conditions. Specifically, waters subject to anthropogenic pressure had a higher conductivity, and a lower color and PAR light intensity (Fig. 9).

**Fig. 10:**
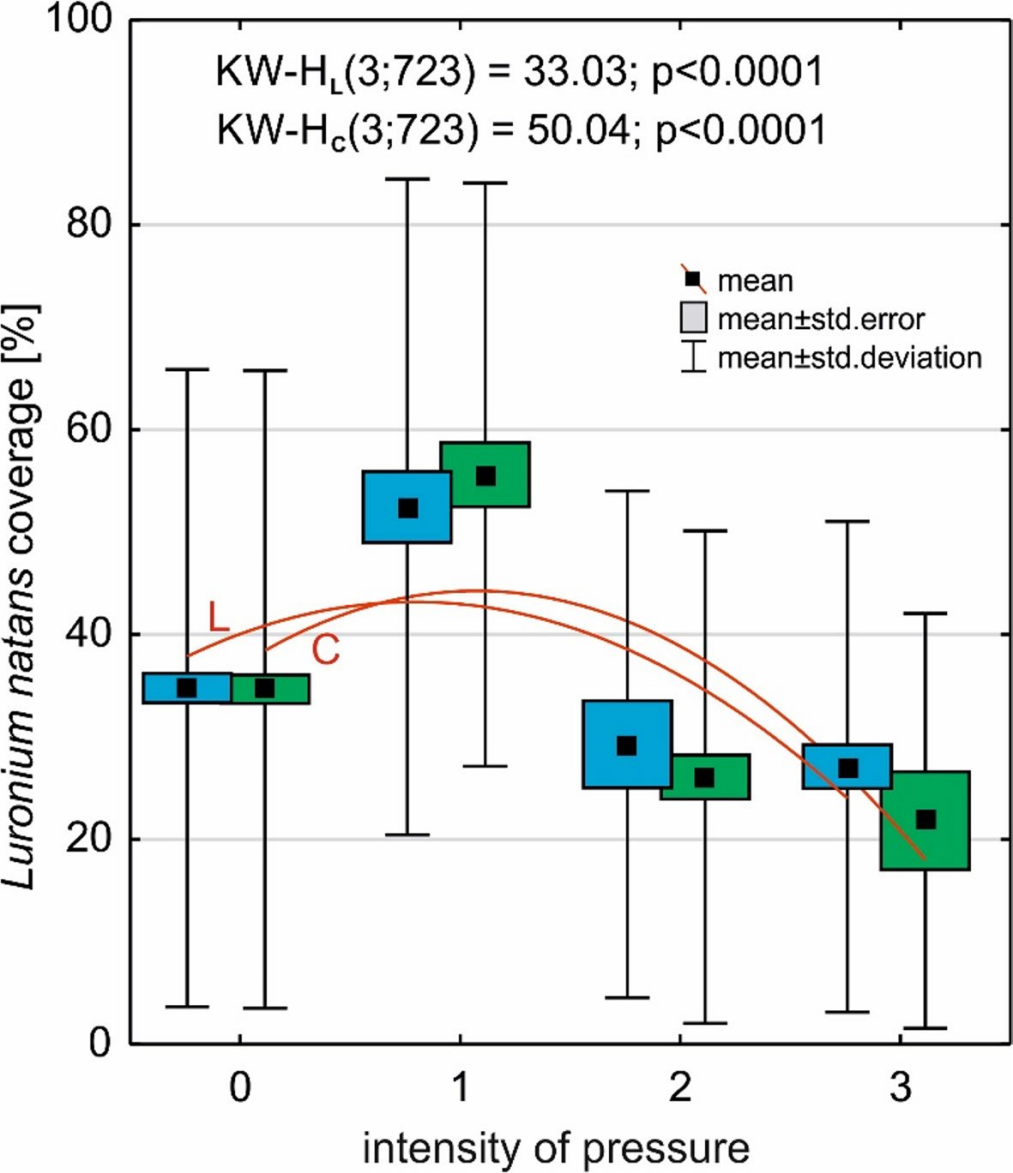
The impact of the intensity of anthropogenic pressure on the cover of *L. natans*. Explanations: L; blue color—the impact on the lake (y = 22.62 + 20.22x-4.97x^2^). C; green color – the impact on the catchment area (y = 20.03 + 24.74x-6.31x.^2^)

A slight increase in anthropogenic pressure intensity led to an increase in the abundance of *L. natans* (Fig. 10); however, a larger pressure resulted in a clear reduction in cover. It should be noted that the impacts on *L. natans* abundance were very similar regarding pressure directed at the lake and its catchment; however, this was slightly larger for catchment transformations.

**Fig. 11:**
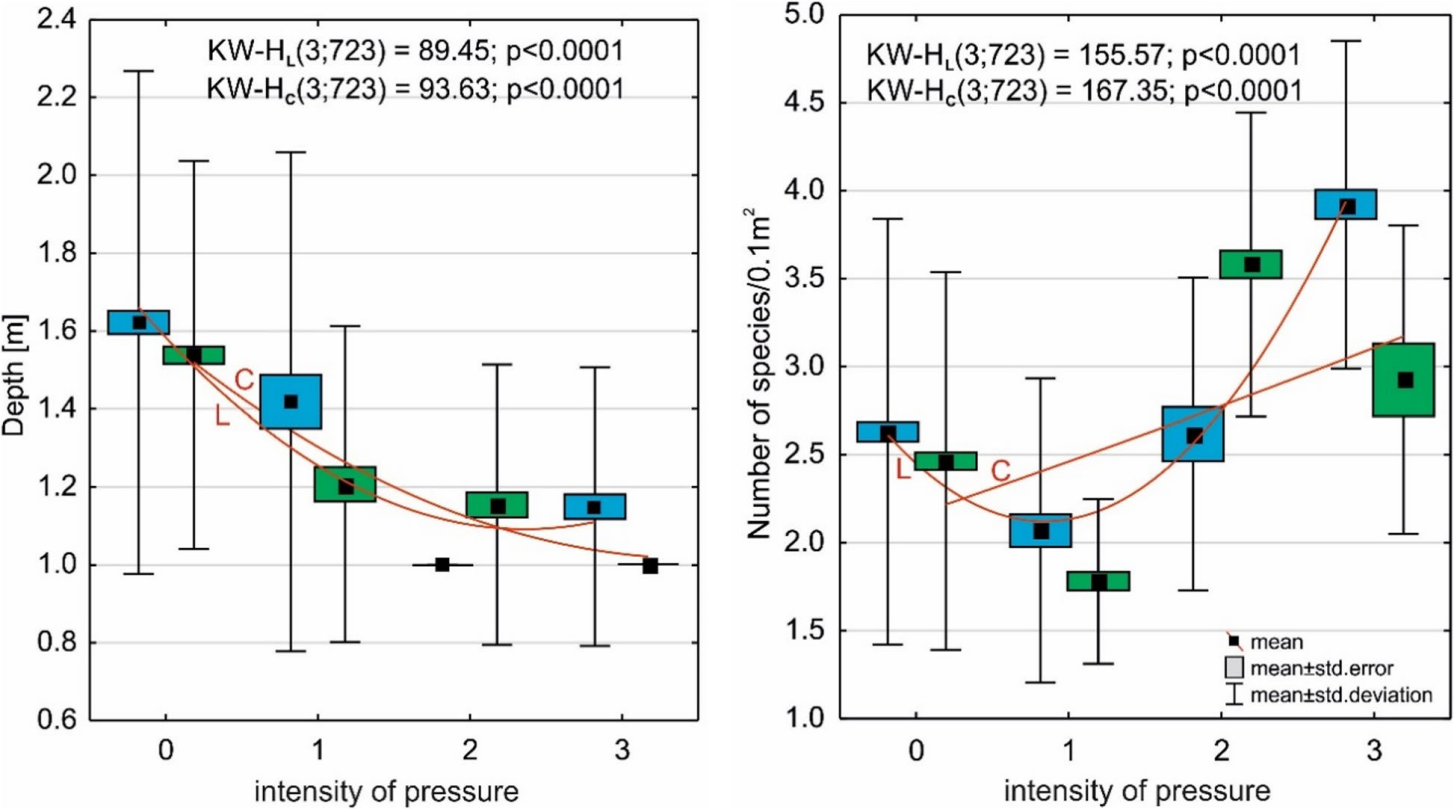
The impact of the anthropogenic pressure on the occurrence depth of plants and the number of species. Explanations: L; blue color—the impact of the lake on the occurrence depth of plants (y = 2.20–0.62x + 0.09x^2^) and the number of species (y = 4.03–1.89x + 0.47x^2^); C; green color—the impact on the catchment area on the occurrence depth of plants (y = 1.87–0.39x + 0.05x^2^) and the number of species (y = 2.04 + 0.32x-0.01x.^2^)

The increase in anthropogenic impact on *L. natans* habitats resulted in the community area shifting towards an increasingly shallow littoral (Fig. 11), while the number of species in the community increased simultaneously, and consequently also the competition from other macrophytes.

**Fig. 12:**
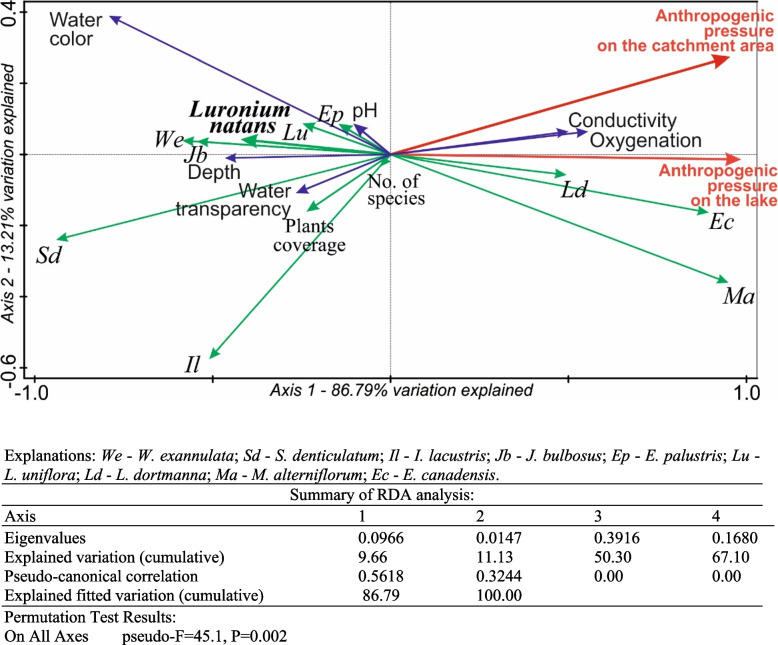
The impact of anthropogenic pressure on environmental conditions and plants abundance based on RDA analysis

The impact of anthropogenic pressure on *L. natans* referred to both the transformation of environmental conditions and community structure (Fig. 12). There is no clear difference in the effects of these transformations regardless of whether the human impact was directed at the lake or its catchment. The intensity of anthropogenic pressure primarily caused an increase in water conductivity and a decrease in water transparency. It is worth emphasizing that, as a result of the pressure, *Luronium* occurred in waters with increasingly less color (despite the decrease in water transparency); this is related to community formation at more shallow depth (cf. Fig. 11), where the water is better oxygenated. The increase in anthropogenic pressure intensity influenced the decrease in the abundance of *L. natans* in the community, but primarily also the decrease in the abundance of other species such as *S. denticulatum* and *I. lacustris*, with a concurrent increase in *M. alterniflorum* and *E. canadensis* cover, as well as *L. dortmanna* to a lesser extent.

## Discussion

*Luronium natans* is a very rare European endemic, although it is found in many different communities, both aquatic and aquatic-terrestrial plants [46]. Literature shows that it is the basic unit of *Littorelletea uniflorae* Br.-Bl. et R.Tx. 1943 class communities [21, 42, 43]. The occurrence of this species was confirmed in patches of *Isoëtetum echinosporae* Koch 1926 em. Dierss. 1975 [39], *Isoëto-Lobelietum* Koch 1926 em. Tx. 1937 [32, 35, 39], *Eleocharitetum multicaulis* (Allorge, 1922) R. Tx. 1937 [17, 39], *Hyperico-Potamogetonetum oblongi* (Allorge, 1921) Br.-Bl. Et Tx. 1952 [39], *Samolo-Littorelletum* Westhoff 1943 [39], *Pilularietum globuliferae* Tx. 1955 ex Müller und Görs 1960 [39], *Eleocharitetum acicularis* (Bauman, 1911) Koch 1926 [17, 21, 39], *Eleocharito-Littorelletum uniflorae* Chouard 1924 [32], and *Myriophyllo-Nupharetum* Koch 1926 [32]. *Luronium* was also found in *Potametea* R. Tx. et Prsg. class communities, *Isoëto-Nanojuncetea* Br.-Bl. Et R. Tx. 1943 class communities [21], and *Phragmitetea* R.Tx. et Prsg. 1942 class communities [47]. It was occasionally found in *Utricularietea intermedio-minoris* Den Hartog et Segal 1964 em. Pietsch 1965 [40, 48, 49] community. Some authors report that *L. natans* forms its own community, namely *Luronietum natantis* Szańkowski ex Šumberová, Čtvrtlíková et Bauer in Chytrý 2011 ass. nova [50], in which it is a characteristic and dominant species. According to Šumberová [50], the *Luronium* community forms within the shallow littoral zone at a depth of between 0.1 to 2.5 m. In Poland, it also occurs at similar depths (1–2 m) [9], although as indicated by our research in Pomeranian lakes, it is occasionally found up to a depth of 3.5 m (cf. Fig. 3, Table 4). In Czech Republic reservoirs it forms a community mainly with *Callitriche hamulata*, *Juncus bulbosus* and *Potamogeton natans* [50]. In Poland, due to harsh winters, *Luronium* mainly forms part of aquatic plant communities, most often in the *Littorelletea uniflorae* Br.-Bl. et R.Tx. 1943 class, and occurs with other isoetids, predominantly *I. lacustris* and *L. dortmanna* (Table 1). It often forms a community with *S. denticulatum*, and slightly less frequently with *M. alterniflorum* and* E. canadensis*. In addition, a common element of this community, as in the Czech Republic, is *J. bulbosus* (11.2%, Table 1), and less frequently *P. natans* (0.2%), but never *C. hamulata*.

**Fig. 13:**
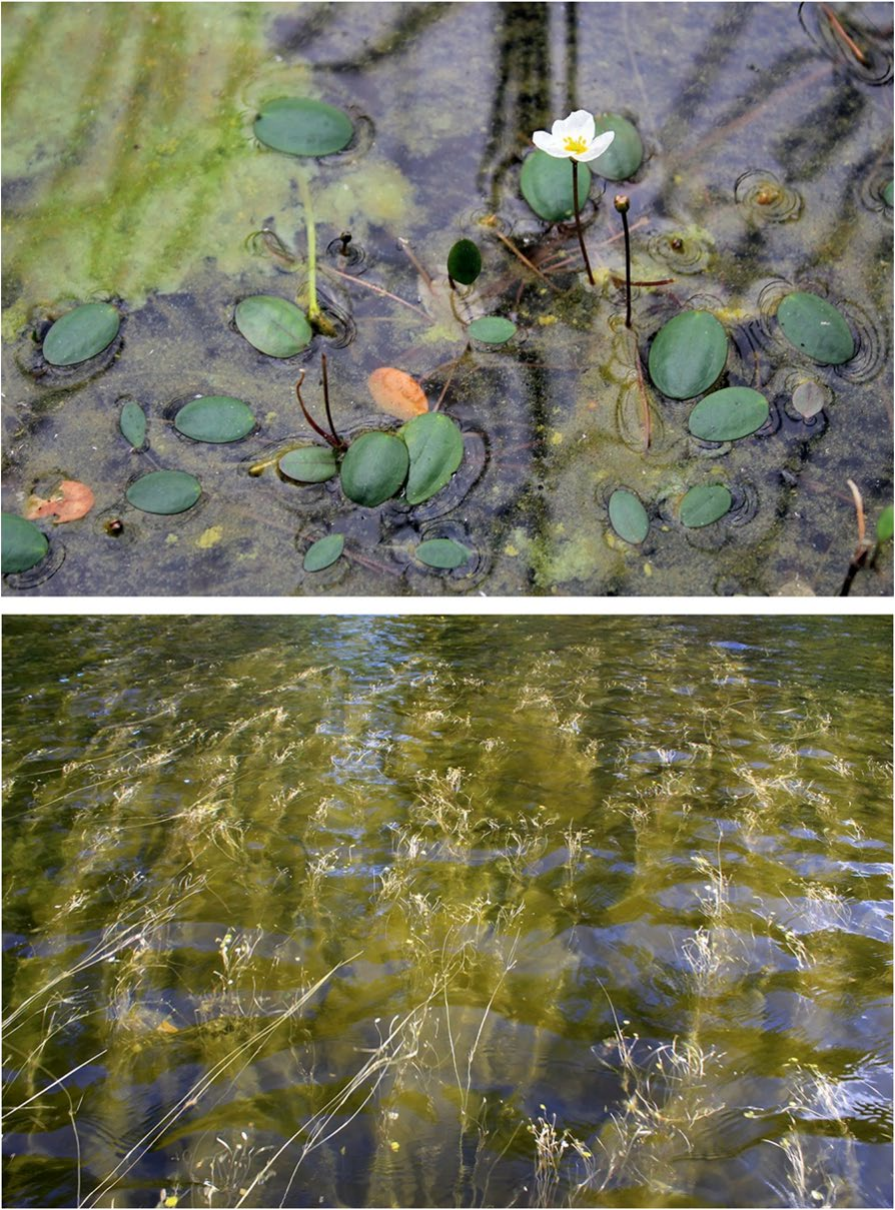
The shallow-water population (top image) and deep-water population (bottom image) of *Luronium natans*

The main factor limiting the development of *Luronium*, and the reason for the decrease in the number of its sites, relates to its intolerance of competition [27, 33], even when resulting from the natural succession of communities. This is also why *Luronium* retreats from anthropogenic habitats, such as ditches and canals subjected to rapid overgrowing [51]. This intolerance may result for several reasons, including physical overgrowth, and competition for light and nutrients. All of these factors likely play a significant role, since they often occur concurrently. This is particularly important in the present, since the pressure on the lakes is very high and the anthropogenic transformations of *Luronium* habitats are significant, for example, as a result of their eutrophication. One of the effects of advanced eutrophication includes mass blooms of plankton algae and the development of filamentous algae, which leads to a decrease in light intensity, as well as transformations in the structures of biocenoses developed by isoetids [52–55]. However, it is worth emphasizing that *Luronium*, as a plant capable of producing surface leaves, is quite resistant to the effects of eutrophication (Fig. 13) and occurs in highly transformed lakes. Floating leaves enable improved access to light, the intensity of which may be low in stained eutrophic water. Floating surface leaves also allow access to atmospheric carbon dioxide, which may be in short supply in eutrophic waters since it is not available at a pH higher than 8.3. *Luronium* prefers free CO_2_ for photosynthesis, but is able to use also bicarbonate [45]. Specimen sizes decrease with increases in trophies, while population densities decrease, and their ranges shift closer towards the shore [9]; however, population reproductive potential is not significantly disturbed. Literature shows that, under conditions of trophic growth, slow-growing isoetids are displaced by faster-growing elodeids [56, 57]. However, *Luronium* growth rates are quite significant for isoetids, so it can be assumed that it is not as easily eliminated from communities as other isoetids. On the other hand, *Luronium* rosettes are less durable compared to other isoetids and decompose much faster, especially in highly disturbed conditions, which primarily applies to underwater leaves.

**Fig. 14:**
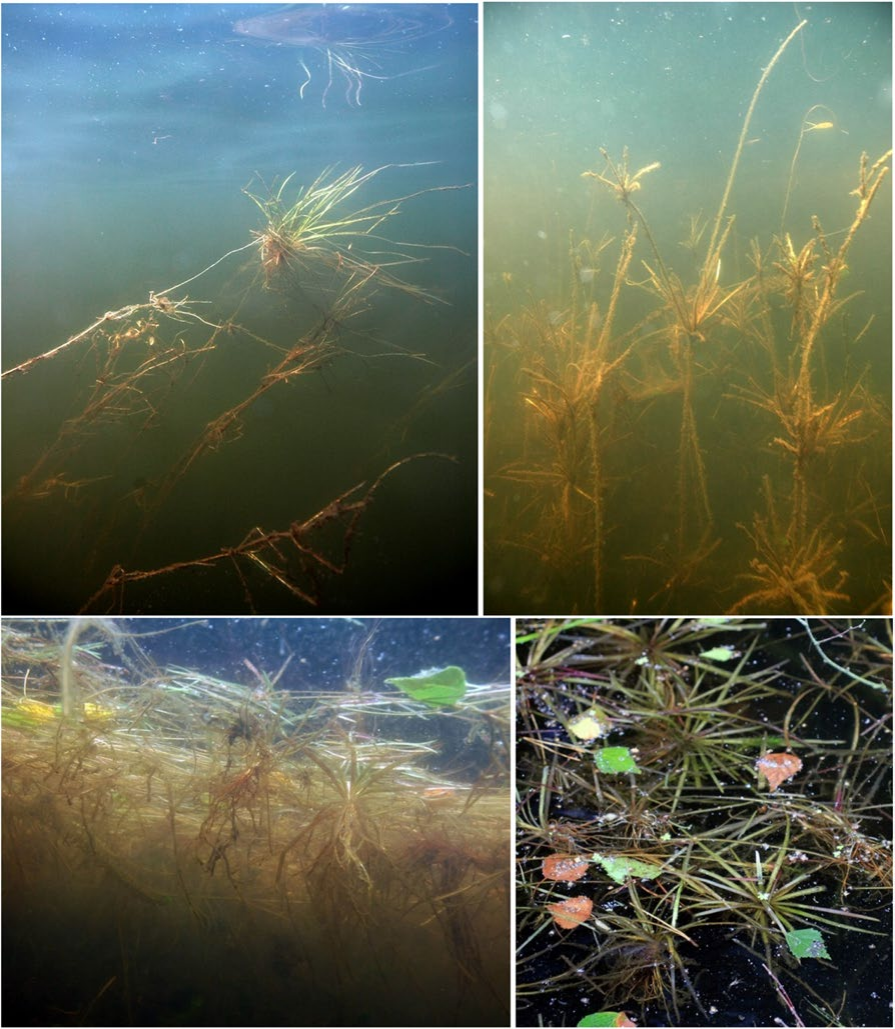
Uprooted individuals of *Luronium natans.* Top photos—for an extended time period uprooted individuals of deep-water populations remain in water due to stolons; Bottom photos—completely uprooted individuals of *Luronium* remain on the water surface among rush plants or with floating leaves where they bloom and fruit

It is also worth noting decreased water levels, resulting from climate change, can contribute to a significant reduction in the typical, and usually very abundant, deep-water populations. Poor rooting in organic sediments causes a mass release and floating up of entire patches of isoetids (Fig. 14), and not only for *Luronium natans*. This may soon contribute to a significant reduction in the abundance of deep-water isoetid populations, including, but not limited to, *Luronium*. Uprooted individuals of deep-water *Luronium natans* populations can remain in water for extended time periods due to stolons. Completely uprooted plants can then remain on the water surface among rush plants, or with floating leaves, where they bloom and fruit or are drifted by wave action to the shore (Fig. 14), where they root again and establish terrestrial populations of floating water-plantain.

Literature indicates that floating water-plantain seems to have a very wide range of tolerance to the physical and chemical properties of aquatic environment [6, 58, 59]. However, this information is often contradictory and differs for local populations. In the Czech Republic *L. natans* occupies habitats with clear and colorless water of pH 6–7, containing small amounts of dissolved substances, especially nitrogen and phosphorus, and up to 30 mg Ca dm^−3^ of calcium [50]. Similar habitats occur in Germany [20]. In Poland, *L. natans* grows in waters with very similar characteristics [9], but mainly in oligotrophic water, with a slightly lower pH of 4.8–6.3, and a very low calcium content (1–2 mg Ca dm^−3^). In the Atlantic part of the area, where *Luronium* has an ecological optimum, it has often been observed in eutrophic waters with very high concentrations of calcium [27] and in waters with an exceptionally wide pH range (3.6–8.0) [6]. This study confirms that *Luronium* is characterized by a large ecological range, but this only applies to a few features of the aquatic environment. For some features, the range of variability for *Luronium* habitats is slightly wider than other isoetids, but it should be assumed that it still occurs in the same habitats as other isoetids (cf. Fig. 8), thereby usually forming a common community. Previous studies [22] have indicated that significant differences exist between *L. natans*, and *I. lacustris* and *L. dortmanna* communities, specifically in their waters, but also in their substrates. Despite its adaptation to adverse conditions (surface leaves), *Luronium* is not found in alkaline waters that are fertile and rich in calcium, as in the case of *L. uniflora* (cf. Additional file 1: Figs. S4-S8). Bazydło [9] reports that high levels of pH (pH > 8.0), carbon (> 6.0 mg C dm^−3^), and phosphorus (> 30.0 µg TP dm^−3^) impede the growth of *Luronium* populations. These specific environmental conditions, and a wide range of variability in *Luronium* habitats, are primarily demonstrated by its occurrence in very poor habitats of dystrophic lakes. Here, floating leaves enable it to compete even with abundant bryophytes of the *Sphagnum* genus. It also allows *Luronium* to survive in waters that are intensely colored by humic substances (cf. Table 2, Fig. 3). *Luronium*’s range of tolerance to water coloration is the largest among isoetids (see Additional file 1: Fig. S6), but it also tolerates lower levels of pH, conductivity, calcium, and trophy (cf. Additional file 1: Figs. S5-S8), which other indicator species of lobelia lakes do not tolerate [60]. This is confirmed by Szańkowski and Kłosowski [22], and indicates a clear preference of *Luronium* for growing in oligotrophic, extremely soft, and calcium-poor waters. Therefore, *Luronium natans* is a species that significantly expands the diversity of habitat 3110 in the Natura 2000 [61], to include mainly dystrophic basins where the remaining isoetids can no longer be found.

## Conclusion

The community structure and environmental conditions of *Luronium* differ distinctly between shallow- and deep-water zones. *Luronium natans* has a broader ecological spectrum than other isoetids due to being abundant in extremely oligotrophic, acidic, and soft waters. Low-intensity anthropogenic pressure focused on a lake or its catchment area leads to an increase in *L. natans* abundance. In turn, a strong anthropogenic pressure on *L. natans* habitats negatively affects both environmental conditions in the lake and the structure of underwater communities, from which *L. natans* regresses or moves towards the shallowest phytolittoral zones.

Our work confirms that *Luronium* occurs mainly with other isoetids, primarily with *I. lacustris* and *L. dortmanna*. It often forms a community only with *S. denticulatum*, and that is why *Luronium* is a species that significantly expands the diversity of habitat 3110 in the Natura 2000 network, to include mainly dystrophic lakes where the remaining isoetids can no longer be found. Therefore, it is worth considering *Luronium* as an indicator species of lobelia lakes because it is the only isoetid in habitats with such advanced natural succession. Dystrophic lakes without *Lobelia*, *Isoëtes*, and *Littorella* are now treated as former lobelia lakes/degraded lobelia lakes, although they still contain a very rare isoetid, namely *Luronium*, which is especially valuable for the preservation of biodiversity in Europe.

## Methods

### Samples collection and environmental analysis

**Fig. 15:**
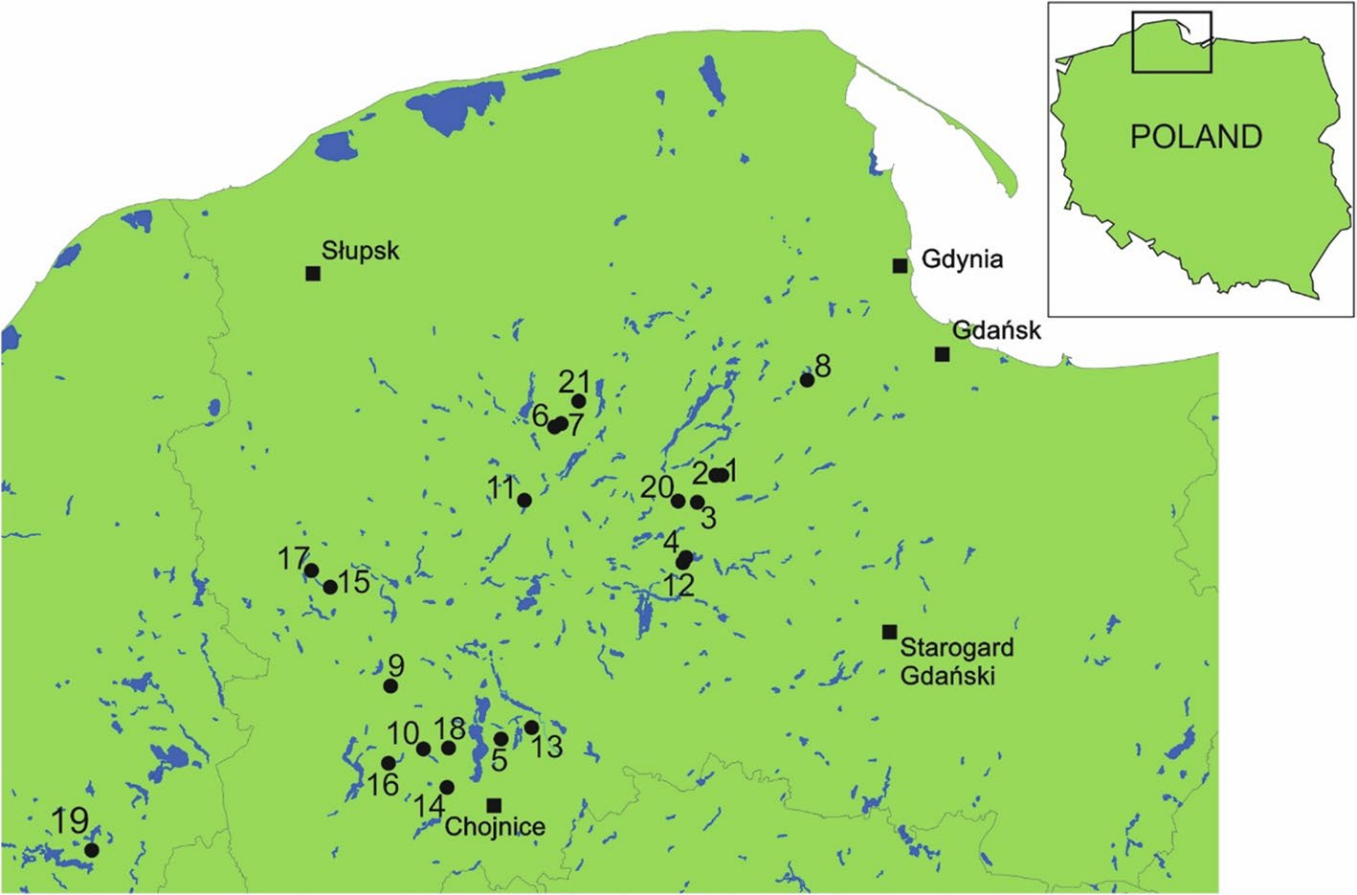
Location of the study sites (1–21; for explanations, see Table 7)

The material for the study was collected each July from 2010 to 2020, from 21 lakes of the Pomeranian Lakeland, where *Luronium natans* is distributed (Fig. 15; Table 7). All studied lakes are low-trophy reservoirs; 18 of them are lobelia lakes with indicator species, and three are dystrophic lakes with *L. natans* and without the presence of indicator species, namely *Lobelia*, *Isoëtes* and *Littorella* (Nos. 1, 2, and 16). Of the lobelia lakes, 11 are nutrient-balanced (typical) and characterized by low trophy, a pH close to neutral, and the usual presence of all indicator species. Two of the surveyed reservoirs were eutrophic lobelia lakes (lakes 8 and 20), distinguished by higher trophy than nutrient-balanced lobelia lakes, a higher pH (alkaline) and a higher water color, and the occurrence of mainly *L. uniflora* and *M. alterniflorum*. The development of these lakes, and the succession of vegetation, are moving towards a eutrophic state. Five of the studied lakes were dystrophic lobelia lakes (5, 6, 7, 9 and 10), characterized by a very acidic pH, brown water color and very low trophy. The indicator species of these lakes are primarily representatives of the genus *Isoëtes*, and a large proportion of *Sphagnum* and *Luronium* exists in their underwater communities. The development of this group of lobelia lakes, and the succession of vegetation, leads to the formation of dystrophic lakes and, consequently, peatbogs.

**Table 7:** Coordinates of the study lakes (1–21), and number of transects and depth zones per lake

No.	Name of lake	Coordinates	No. of tnasects	No. of depth zones	No. of zones with *Luronium*
1	Nameless 1	54°11′30.8"N 18°05′55.8"E	1	2	2
2	Nameless 2	4°11′25.4"N 18°05′53.4"E	1	2	2
3	Dobrogoszcz	54°08′46.6"N 18°02′09.4"E	7	6/6/6/6/6/5/3	1
4	Drzędno	54°03′47.2"N 18°00′34.1"E	1	3	1
5	Gacno Wielkie	53°47′36.1"N 17°33′35.3"E	2	5/4	1
6	Jelenie Małe	54°15′10.9"N 17°40′53.3"E	1	2	1
7	Jelenie Wielkie	54°15′22.5"N 17°41′16.6"E	2	7/5	1
8	Karlikowskie	54°19′25.5"N 18°18′21.7"E	2	5/2	1
9	Krasne	53°52′03.1"N 17°16′54.3"E	3	5/5/2	2
10	Linówko	53°46′40.5"N 17°21′53.8"E	2	3/2	1
11	Łąkie	54°08′48.3"N 17°36′18.8"E	2	12/ 7	1
12	Małe Oczko	54°03′20.6"N 18°00′13.4"E	1	3	1
13	Moczadło	53°48′51.1"N 17°38′00.9"E	4	11/10/10/6	2
14	Okoń Duży	53°43′16.8"N 17°25′39.9"E	1	4	1
15	Piasek	54°00′39.4"N 17°07′31.2"E	3	13/10/8	2
16	Pijawka	53°45′19.5"N 17°16′53.0"E	1	3	1
17	Smołowe	54°01′58.3"N 17°04′38.6"E	4	10/8/3/3	4
18	Sporackie	53°46′43.2"N 17°25′48.8"E	1	3	1
19	Śmiadowo	53°37′01.6"N 16°33′09.4"E	2	10/8	1
20	Świniebudy	54°08′49.5"N 17°59′22.0"E	1	5	1
21	Warleńskie	54°17′28.3"N 17°44′08.7"E	1	2	1
Total:			43	241	29

The samples were collected using the diving method, in transects perpendicular to the lakeside (as per the methodology of Chmara et al. [62]). Diving was performed within the phytolittoral zone of each lake, at depths ranging from the shallowest zone (0.5 m) to the lower limit of plant occurrence. In total, the research was conducted in 241 depth zones within 43 transects, each being approximately 250 m wide (Table 7). In each zone (a bottom strip at 1.0 m intervals), a non-invasive diving method was applied to describe an average of about 50 random plant samples. Phytosociological relevés were taken, each with an area of 0.1 m^2^. Total plant cover was determined for each sample. A total of 8 732 plant samples were described from the lakes, with *Luronium* occurring in 723 samples. Only 34 samples (4.7%) had *Luronium* cover < 5%, and only 23 had a cover of 1%, which were samples at the edges of community ranges. In such samples, *Luronium* usually occurred with a low cover, but often without the contribution of other species.

Environmental condition assessments were performed based on the samples of near-sediment water and sediments from depth zones. Field measurements were made in the depth profile from a pontoon anchored in the deepest part of the lake. From each depth zone where the plant samples were described, three samples of near-sediment water (approx. 5 cm above the bottom, where plants grow) were collected, with a volume of 0.5 dm^3^, and three samples of sediment of similar volume were taken in the area where plants are rooted. Water and sediment samples were taken at the beginning of each littoral zone (depth zone), as well as the middle and at the end. Samples were taken by the diver at the beginning of the underwater work, and were allowed to determine the maximum extent of submerged plants, as well as dive to plants in each zone. Plant samples were generally taken starting from the deepest zone, then from successively more shallow zones, and finally from the shallowest, to avoid disturbing the environment by the diver. In total, 543 water and sediment samples, respectively, were collected in the analyzed lakes, with 87 water samples and 87 sediment samples from 29 depth zones for the analyses of physical and chemical characteristics of *Luronium* habitats. Water samples were analyzed as per the methods proposed by Eaton et al. [63]. Water pH, conductivity, concentration of calcium, CO_2_, HCO_3_^−^, nitrogen, phosphorus and humic acids, and color were determined, whereas in the depth profile, water oxygenation, temperature, and intensity of photosynthetically active radiation (PAR) were measured in the field. In sediments, pH, redox potential, and electrolytic conductivity were measured, and the content of organic and mineral matter, as well as sediment hydration, were determined. Sampling depths were determined using the Eagle TriFinder depth finders, and water transparency by a Secchi disk without a glass pane on water surface. Water and sediment pH were measured using a pH-meter 320/SET1 with a SENTIX 97 T electrode. Water oxygenation and temperature were measured by a WTW OXI 197 oxygen meter with an EOT 196 electrode. PAR light intensity were measured by a Licor LI–250 Light Meter, and given as a percentage of the light reaching the water surface. Calcium concentration was determined with complexometric methods. Humic acid concentration was determined spectrophotometrically using a UV–VIS Aquamate spectrophotometer at 330 nm. The concentration of dissolved forms of inorganic carbon (CO_2_ and HCO_3_^−^) was assessed in the collected water samples by titration, using phenolphthalein and methyl orange, respectively. Water color was determined with a comparative method using the Platinum-Cobalt Reference Standards. Finally, total nitrogen and phosphorus were determined using Merck Spectroquant Cuvette Tests.

Lake pressure was determined on a scale of 0–3, where 0 represents no pressure (e.g., protection in lake reserves), 1 represents a low pressure (e.g., amateur fishing), 2 represents a medium intensity (e.g., intensive fishing, poorly developed recreation), and 3 represents intense pressure (fishing management, recreation, inflow of drainage waters from drained bog habitats, etc.). The pressure on the lake catchment was also determined on a scale of 0–3; where 0 represents no pressure (the catchment is natural, forest), 1 represents a low pressure (small transformations in the catchment, small deforestations), 2 represents medium transformations in the catchment (significant deforestation, mainly grassland, small areas of arable land), and 3 represents large catchment transformations (arable land dominant, urban, rural and summer buildings).

### Statistical methods

The data collected on the environmental conditions and vegetation of individual lakes was catalogued in Microsoft Excel. Each entry recorded the number of species and the related plant cover, and all samples from a given depth zone were assigned to appropriately characterized environmental conditions, i.e., the average of the three water and sediment samples collected in each depth zone.

A total of 38 macrophyte species were found in the lakes analyzed. Average cover and frequency were determined for each species, as well as basic cover statistics (Table 1). All samples with *L. natans* (723) were used for further analysis of the community structure and its environmental conditions.

Community structure was described to verify the first hypothesis, and nine species with the highest frequency (> 5%, Fig. 1) were distinguished and further analyzed. Differences in *L. natans* cover in patches with these species were also determined (Fig. 2) using a nonparametric Kruskal–Wallis test. To indicate how *Luronium* cover changes with depth gradient, a Kruskal–Wallis test (Fig. 4) and HSD Tukey's post hoc test for unequal abundances (Table 3) were performed, while statistically significant differences between the community structure of shallow- and deep-water populations of *Luronium* were performed using the Mann–Whitney U test (Tables 4 and 5).

The environmental conditions of *L. natans* in the studied lakes were described to verify the second hypothesis (Table 2). Differences in habitat patches formed by *Luronium* with the highest frequency species (9 species with F > 5%, Additional file 1: Figs. S1-S3) were also determined using the non-parametric Kruskal–Wallis test. In addition, the rank of environmental factors in the formation of these *Luronium* patches (Fig. 3) was determined using Canonical-correlation analysis (CCA) in the CANOCO 5.1 software [64]. Statistically significant differences between environmental conditions of the shallow- and deep-water *Luronium* populations were determined using the Mann–Whitney U test (Table 5) using Statistica 13.1.

The two hypotheses were also verified by distributing shallow- and deep-water patches of *Luronium*, taking into account both the coverages of all species and the environmental features studied, using Principal Component Analysis (PCA) in CANOCO 5.1 (Fig. 7).

To verify the third hypothesis, we used the results from our underwater plant database AquaPlant, containing 8,662 samples of *I. lacustris*, 4,022 samples of *L. dortmanna*, and 2,214 samples of *L. uniflora*. Differences in environmental conditions of *Luronium* and other isoetids were also determined (see Additional file 1: Figs. S4-S8). The habitat distribution of *L. natans* and other isoetids was determined using PCA analysis based on species occurrence and environmental variables (Fig. 8). Calculations were performed in PAST v. 4.05 based on the correlation matrix.

To determine the relationship between the structure of the *Luronium* community and environmental conditions, and the intensity of anthropogenic pressure (the fourth hypothesis), the following were determined: (1) Spearman's rank correlation—correlation pressure in the lake and catchment on species depth, number of species, and coverage of *Luronium*, and total plant number (Table 6); (2) the effect of anthropogenic pressure intensity on *L. natans* habitat and coverage using the non-parametric Kruskal–Wallis test (Figs. 9, 10 and 11); and (3) the effect of anthropogenic pressure on environmental conditions and plant abundance based on Redundancy analysis (RDA; Fig. 12) in CANOCO 5.1 [64].

### Supplementary Information

**Additional file 1:** **Figure S1.** Water conditions in the *Luronium natans* community with species with a frequency > 5%. Explanations: 1- *I. lacustris*, 2- *L. dortmanna*, 3- *S. denticulatum*, 4- *M.* *alterniflorum*, 5-*J. bulbosus*, 6- *E. canadensis*, 7- *W. exannulata*, 8-*L. uniflora*. 9- *E. palustris*. **Figure S2.** Oxygenation and water temperature, PAR light intensity and water transparency in the *Luronium* community with species with a frequency > 5%. Explanations see Fig. S1. **Figure S3.** Environmental conditions in the sediment in *Luronium* community with species with a frequency > 5%. Explanations see Fig. S1. **Figure S4.** Differences in the depth of occurrence, PAR intensity, water oxygenation and temperature between habitats of *L. natans* (*Ln*) and other isoetids (*Il *- *I. lacustris*, *Ld* - *L. dortmanna* and *Lu* - *L. uniflora*). Statistically significant differences were determined with *p*<0.001 - ***, *p*<0.01- **, *p*<0.05 - *. **Figure S5.** Difference in pH, redox potential, calcium concentration and water conductivity between habitats of *L. natans* (*Ln*) and other isoetids (*Il *- *I. lacustris*, *Ld* - *L. dortmanna* and *Lu* - *L. uniflora*). Statistically significant differences were determined with *p*<0.001 - ***, *p*<0.01- **, *p*<0.05 - *. **Figure S6.** Differences in water transparency and color, nitrogen and phosphorus concentration between habitats of *L. natans* (*Ln*) and other isoetids (*Il -* *I. lacustris*, *Ld* - *L. dortmanna* and *Lu* - *L. uniflora*). Statistically significant differences were determined with *p*<0.001- ***, *p*<0.01 - **, *p*<0.05 - *. **Figure S7.** Differences in pH, redox potential, calcium concentration and sediment conductivity between habitats of *L. natans* (*Ln*) and other isoetids (*Il *- *I. lacustris*, *Ld* - *L. dortmanna* and *Lu *- *L. uniflora*). Statistically significant differences were determined with *p*<0.001 - ***, *p*<0.01- **, *p*<0.05 - *. **Figure S8.** Differences in nitrogen and phosphorus concentration, organic matter and sediment hydration between habitats of *L. natans* (*Ln*) and other isoetids (*Il *-*I. lacustris*, *Ld* - *L. dortmanna* and Lu - *L. uniflora*). Statistically significant differences were determined with *p*<0.001 - ***, *p*<0.01- **, *p*<0.05 - *.

## Data Availability

The datasets used and/or analysed during the current study are available from the corresponding author on reasonable request.
