# Supplementary material for: Community structure, environmental conditions and anthropogenic pressure on the habitat of the European endemic aquatic plant Luronium natans (L.) Raf
Source: BMC Plant Biol. 2023 Nov 28;23:596. doi: 10.1186/s12870-023-04518-y (PMC10683176; doi:10.1186/s12870-023-04518-y)
Supplement: Supplementary file 1 — Additional file 1: Figure S1. Water conditions in the Luronium natans community with species with a frequency > 5%. Explanations: 1- I. lacustris, 2- L. dortmanna, 3- S. denticulatum, 4- M. alterniflorum, 5-J. bulbosus, 6- E. canadensis, 7- W. exannulata, 8-L. uniflora. 9- E. palustris. Figure S2. Oxygenation and water temperature, PAR light intensity and water transparency in the Luronium community with species with a frequency > 5%. Explanations see Fig. S1. Figure S3. Environmental conditions in the sediment in Luronium community with species with a frequency > 5%. Explanations see Fig. S1. Figure S4. Differences in the depth of occurrence, PAR intensity, water oxygenation and temperature between habitats of L. natans (Ln) and other isoetids (Il - I. lacustris, Ld - L. dortmanna and Lu - L. uniflora). Statistically significant differences were determined with p<0.001 - ***, p<0.01- **, p<0.05 - *. Figure S5. Difference in pH, redox potential, calcium concentration and water conductivity between habitats of L. natans (Ln) and other isoetids (Il - I. lacustris, Ld - L. dortmanna and Lu - L. uniflora). Statistically significant differences were determined with p<0.001 - ***, p<0.01- **, p<0.05 - *. Figure S6. Differences in water transparency and color, nitrogen and phosphorus concentration between habitats of L. natans (Ln) and other isoetids (Il - I. lacustris, Ld - L. dortmanna and Lu - L. uniflora). Statistically significant differences were determined with p<0.001- ***, p<0.01 - **, p<0.05 - *. Figure S7. Differences in pH, redox potential, calcium concentration and sediment conductivity between habitats of L. natans (Ln) and other isoetids (Il - I. lacustris, Ld - L. dortmanna and Lu - L. uniflora). Statistically significant differences were determined with p<0.001 - ***, p<0.01- **, p<0.05 - *. Figure S8. Differences in nitrogen and phosphorus concentration, organic matter and sediment hydration between habitats of L. natans (Ln) and other isoetids (Il -I. la [file 12870_2023_4518_MOESM1_ESM.docx]

Fig. S1. Water conditions in the *Luronium natans* community with species with a frequency > 5%. Explanations: 1- *I. lacustris*, 2- *L. dortmanna*, 3- *S. denticulatum*, 4- *M.* *alterniflorum*, 5- *J. bulbosus*, 6- *E. canadensis*, 7- *W. exannulata*, 8-*L. uniflora*. 9- *E. palustris*.

Fig. S2. Oxygenation and water temperature, PAR light intensity and water transparency in the *Luronium* community with species with a frequency > 5%.

Explanations see Fig. A.1.

Fig. S3. Environmental conditions in the sediment in *Luronium* community with species with a frequency > 5%.

Explanations see Fig. A.1.


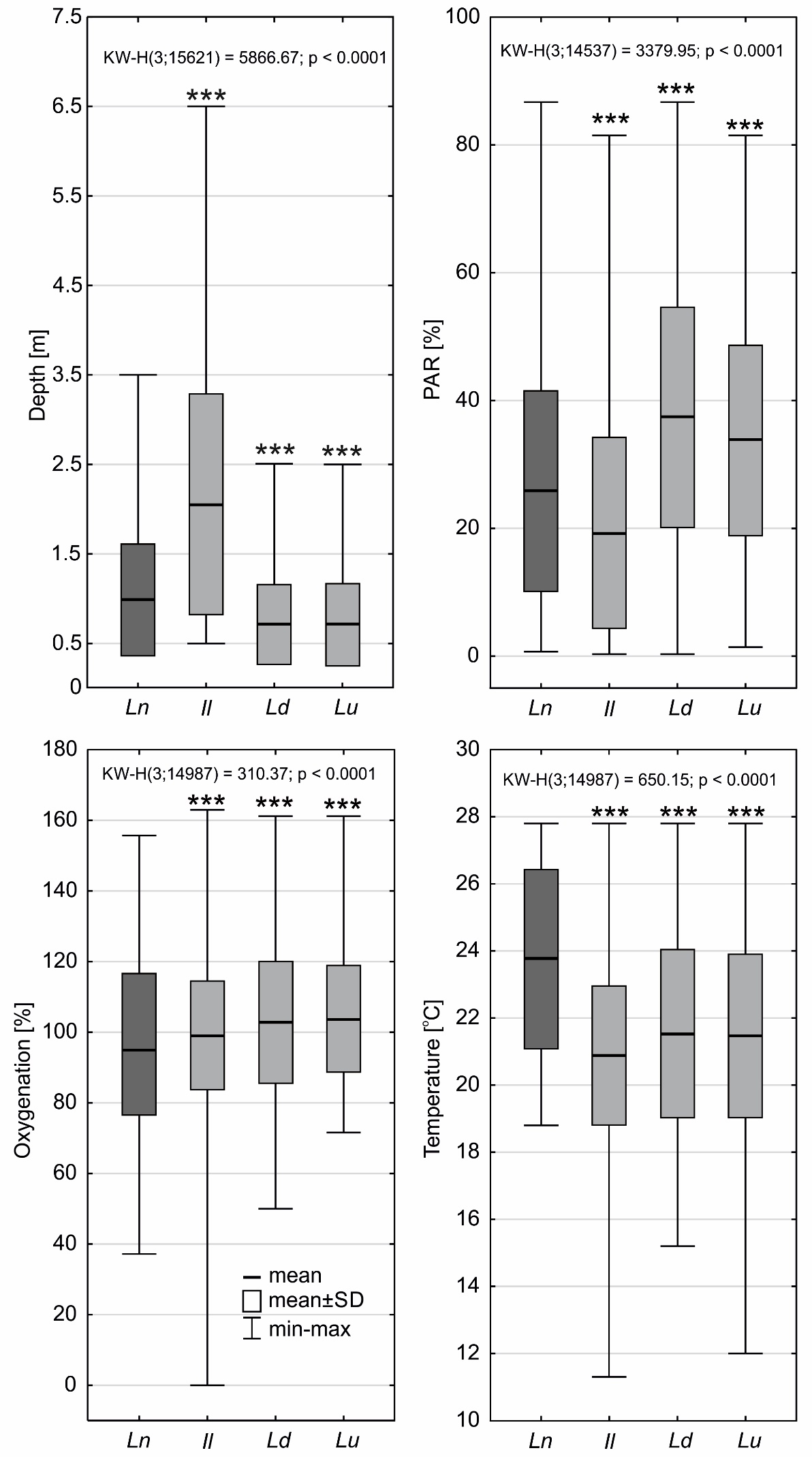


Fig. S4. Differences in the depth of occurrence, PAR intensity, water oxygenation and temperature between habitats of *L. natans* (*Ln*) and other isoetids (*Il* - *I. lacustris*, *Ld* - *L. dortmanna* and *Lu* - *L. uniflora*). Statistically significant differences were determined with p<0.001 - ***, p<0.01 - **, p<0.05 - *.


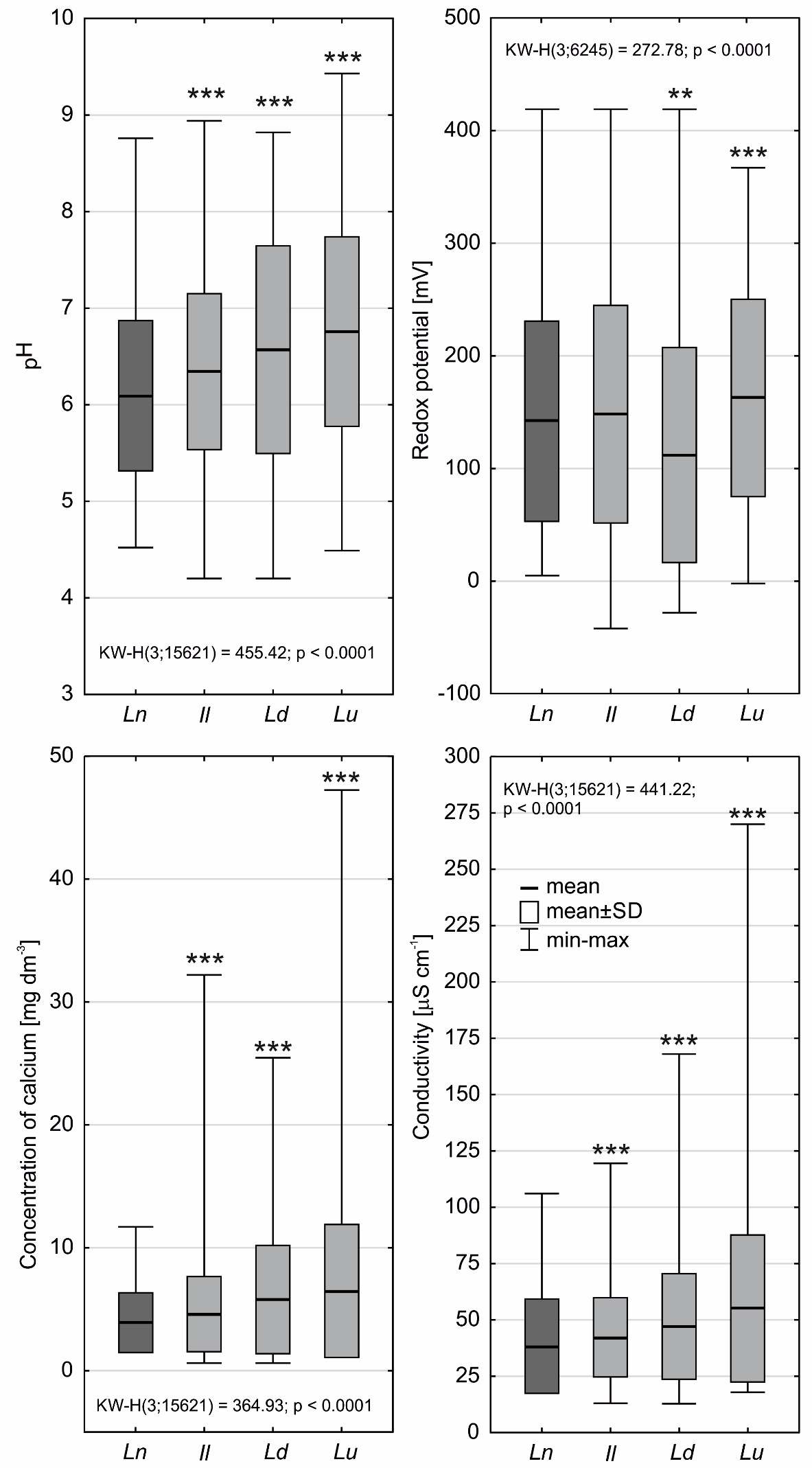


Fig. S5. Difference in pH, redox potential, calcium concentration and water conductivity between habitats of *L. natans* (*Ln*) and other isoetids (*Il* - *I. lacustris*, *Ld* - *L. dortmanna* and *Lu* - *L. uniflora*). Statistically significant differences were determined with p<0.001 - ***, p<0.01 - **, p<0.05 - *.


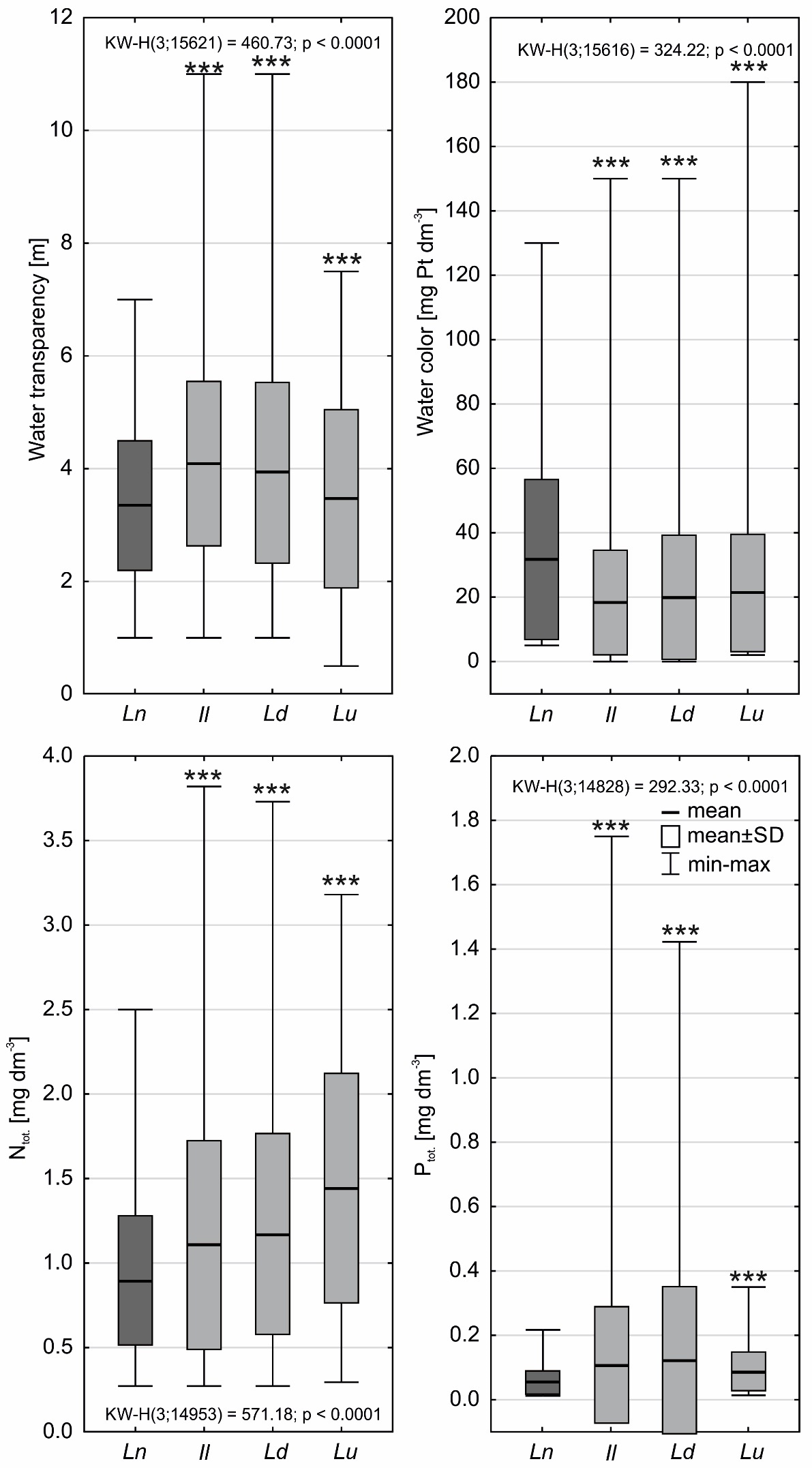


Fig. S6. Differences in water transparency and color, nitrogen and phosphorus concentration between habitats of *L. natans* (*Ln*) and other isoetids (*Il -* *I. lacustris*, *Ld* - *L. dortmanna* and *Lu* - *L. uniflora*). Statistically significant differences were determined with p<0.001 - ***, p<0.01 - **, p<0.05 - *.


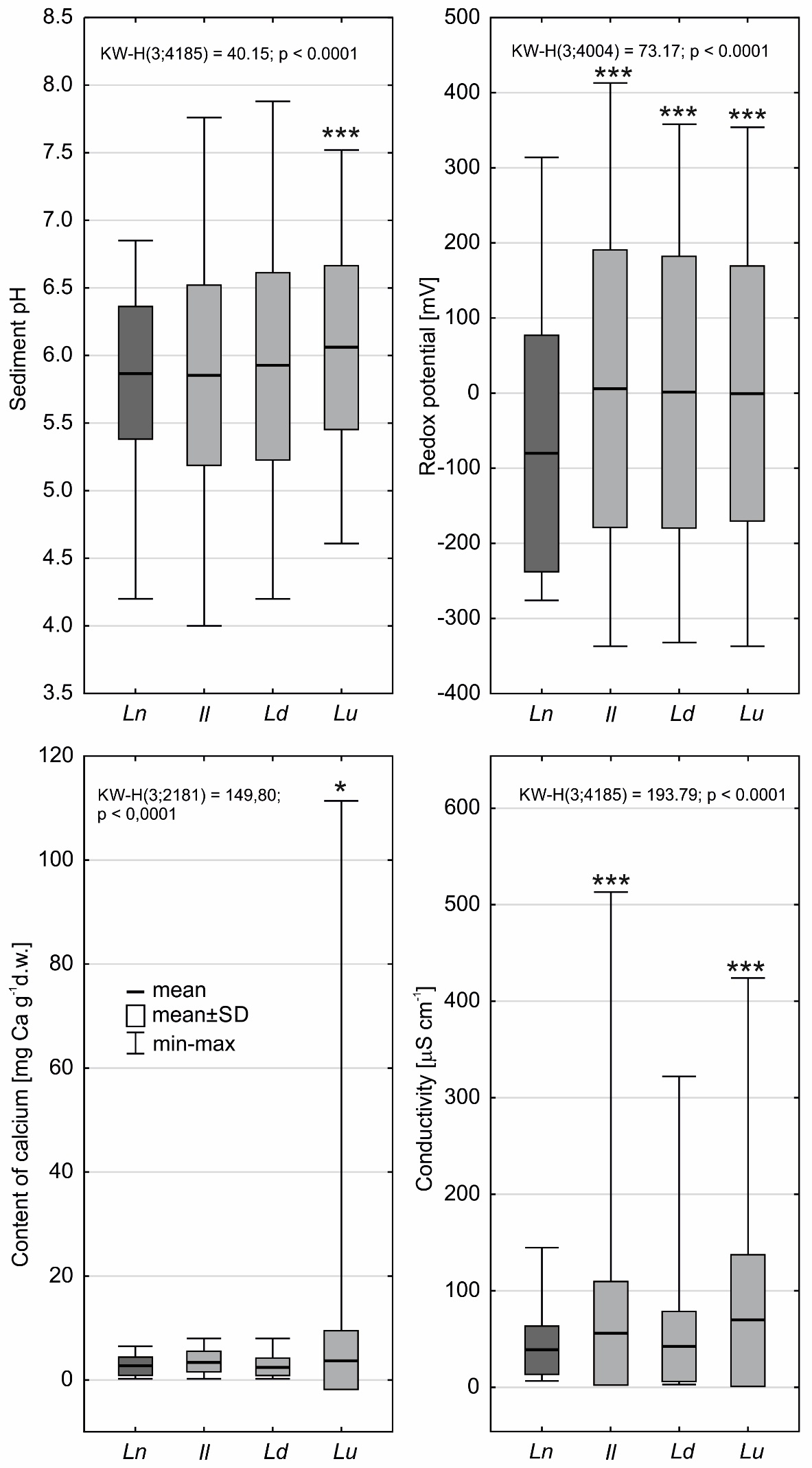


Fig. S7. Differences in pH, redox potential, calcium concentration and sediment conductivity between habitats of *L. natans* (*Ln*) and other isoetids (*Il* - *I. lacustris*, *Ld* - *L. dortmanna* and *Lu* - *L. uniflora*). Statistically significant differences were determined with p<0.001 - ***, p<0.01 - **, p<0.05 - *.

Fig. S8. Differences in nitrogen and phosphorus concentration, organic matter and sediment hydration between habitats of *L. natans* (*Ln*) and other isoetids (*Il* - *I. lacustris*, *Ld* - *L. dortmanna* and Lu - *L. uniflora*). Statistically significant differences were determined with p<0.001 - ***, p<0.01 - **, p<0.05 - *.
